# The *Hydractinia echinata* Test-System. III: Structure-Toxicity Relationship Study of Some Azo-, Azo-Anilide, and Diazonium Salt Derivatives

**DOI:** 10.3390/molecules19079798

**Published:** 2014-07-08

**Authors:** Sergiu Adrian Chicu, Melania Munteanu, Ioana Cîtu, Codruta Şoica, Cristina Dehelean, Cristina Trandafirescu, Simona Funar-Timofei, Daniela Ionescu, Georgeta Maria Simu

**Affiliations:** 1 Institute of Chemistry Timisoara of the Romanian Academy, B-dul Mihai Viteazul 24, RO-300223 Timişoara, Romania; E-Mails: lchi@gmx.de (S.A.C.); timofei@acad-icht.tm.edu.ro (S.F.-T.); 2 Department of Clinical Laboratory and Sanitary Chemistry, “Vasile Goldis” University, 1 Feleacului Str., Arad 310396, Romania; 3 Faculty of Medicine, “V. Babes” University of Medicine and Pharmacy, 2 Eftimie Murgu, 300041 Timisoara, Romania; 4 Faculty of Pharmacy, “V. Babes” University of Medicine and Pharmacy, 2 Eftimie Murgu, 300041 Timisoara, Romania; E-Mails: codrutasoica@umft.ro (C.S.); cadehelean@umft.ro (C.D.); trandafirescu.cristina@umft.ro (C.T.); ionescu.daniela@umft.ro (D.I.); simu.georgeta@umft.ro (G.M.S.)

**Keywords:** *Hydractinia echinata (H. echinata)*, azo derivatives, simple tautomery (ST), double alternante tautomery (DAT), hydrolysis/reduction mechanisms, isotoxicity

## Abstract

Structure-toxicity relationships for a series of 75 azo and azo-anilide dyes and five diazonium salts were developed using *Hydractinia echinata* (*H. echinata*) as model species*.* In addition, based on these relationships, predictions for 58 other azo-dyes were made. The experimental results showed that the measured effectiveness Mlog(1/MRC_50_) does not depend on the number of azo groups or the ones corresponding to metobolites, but it is influenced by the number of anilide groups, as well as by the substituents’ positions within molecules. The conformational analysis pointed out the intramolecular hydrogen bonds, especially the simple tautomerization of quinoidic (ST_OH_) or aminoidic (ST_NH2_) type. The effectiveness is strongly influenced by the “push-pull” electronic effect, specific to two hydroxy or amino groups separated by an azo moiety (double alternate tautomery, (DAT), to the –COOH or –SO_3_H groups which are located in *ortho* or *para* position with respect to the azo group. The levels of the lipophylic/hydrophilic, electronic and steric equilibriums, pointed out by the Mlog(1/MRC_50_) values, enabled the calculation of their average values Clog(1/MRC_50_) (“*Köln model*”), characteristic to one derivative class (class isotoxicity). The azo group reduction and the hydrolysis of the amido/peptidic group are two concurrent enzymatic reactions, which occur with different reaction rates and mechanisms. The products of the partial biodegradation are aromatic amines. No additive or synergic effects are noticed among them.

## 1. Introduction

The importance of azo derivatives is due to broad range of applications, which practically cover all human activities, both as dyes [1] and pigments [2]. In the medical-pharmaceutics field, the use of azo-derivatives is of interest to assess the permeability of the blood-brain barrier (BBB) to macromolecules [3]. Several affections, such as Prion-, Alzheimer’s-, Chorea major- or Parkinson’s diseases, are due to protein misfolding and aggregation (amiloide) processes [4]. The azoic dye Congo Red detects and interfere with their formation process, stabilizes native protein monomers or partially folded intermediates, but generally little is known about the exact mechanism of the inhibitor’s action on protein aggregation [5].

From a structural point of view, an azo derivative molecule contains an azo group –N=N– (chromophore), which binds together two substituted aromatic nuclei. The coplanarity of the dye molecule allows an extended conjugation of the π-electrons of the azo group, –N=N– and of the aromatic nucleus with the p electrons of some +M effect auxochromes (–OH, –OR, –NH_2_, –NR_2_) or of some –M effect antiauxochromes (–NO_2_, –COOH, –SO_3_H) [6].

The main metabolic process to which the azo dyes are subjected in the human organism is the reduction of the azo group. In the first stage, a hydrazo derivative appears, by the action of the electron-withdrawing substituents, such as the sulfonic groups, even when these groups are more separated from the azo group [1]. The second step, e.g., the decomposition of the hydrazo derivative to its corresponding free amines, is dependent on the charge difference among nitrogen atoms, as a direct consequence of some electron-donating substituents [7].

Most azo derivatives contain, beside the azo group, one or more amidic (anilidic) groups (chromophores). The hydrolysis of these compounds occurs enzymatically [8], like the naphthols AS’s hydrolysis [9], involving the formation of an oxyanionic intermediate during the rate-determining step.

The reduction of the azo group and the hydrolysis of an amido group are two different enzymatic reactions, which probably occur concurrently. The biodegradation rate will be given by the slowest step of the reaction and is affected when the coplanarity of the molecule is disturbed by steric hindrances [10].

The reaction products are aromatic amines [11], some of them exhibiting mutagenic effects even via skin microflora, according to *in vitro* experiments with *Staphylococus aureus* [12]. Although the mutagenic or the carcinogenic character may be significantly decreased by sulphonation [13], the presence of the strongly polar sulfonic groups, confers to the molecules a marked hydrophilicity and an increased solubility, reduces very much the permeability of the lipophilic cellular membrane [14] and consequently their biodegradability is decreased to a great extent [15].

The first aim of the present work was the comparative determination of the toxicity of some azo, azo- anilide, and diazonium salts using the biological *H. echinata* test-system (*He*TS). The larvae of *H. echinata* have an elongated spindle shaped body of about 1 mm in length and a diameter of 100 µm. They consist of about 10,000 cells. They have no mouth, no gut, no extremities, and no sense organs other than nerve cells which may serve to sense environmental signals. As a consequence, one could estimate a xenobiotic’s direct action at the cellular level. This matter of fact could be also the reason why the test system proved a higher sensibility for the identification of the toxicity of some nonyl- phenolic isomers in comparison to the Daphnia-immobilisation test, as well as to the duckweed-growth inhibition test [16].

*H. echinata* represent more than substitutes for tests on superior organisms. Further, the influence of structural modifications on effectiveness, the presentation of some hydrolysis/reduction reaction mechanisms of azo-anilide derivatives, and some prediction possibilities for a series of non-tested yet derivatives, were taken into account. Conformational analysis was performed by molecular mechanics and quantum chemical calculations in order to obtain the title compound intramolecular hydrogen bonds, especially the simple tautomerization of quinoidic (ST_OH_) or aminoidic (ST_NH2_) type.

## 2. Results and Discussion

In Table 1 the logarithm of the reciprocal value (log1/MRC_50_) values are presented. The average (C) concentrations of these values were calculated too. The MRC_50_ value calculations (Chicu *et al.* [17]) result from the graphical representation of the metamorphosis variation, M (%), (Y axis) function of the xenobiotic’s concentration (mol/L) (X axis), where the metamorphosis decreases with the rise of the xenobiotic’s concentration. Thus, the MRC_50_ value represents the xenobiotic’s concentration (mol/L) necessary for a 50% decrease of metamorphosis, with respect to control.

The Clog(MRC_50_) value was obtained as the sum of the least squares differences between the Mlog(MRC_50_) measured values and Clog(MRC_50_) (Microsoft Office Excel 2003) based on the experimental data/algorithm according to which +/− 0,50 log.u differences characterize the xenobiotics with identical toxicity (isotoxicity). The accuracy of experimental (log1/MRC_50_) data was checked by the standard deviations, which were determined by the Excel 2003 program. For each concentration of substance experiments were performed in triplicate and were repeated at least twice.

**Table 1:** *Hydractinia echinata* test system: experimental Mlog/(1/MRC_50_) (M) and calculated average Clog(MRC_50_) (C) toxicities of some azo-, azo-anilide, diazonium salts derivatives.

No.	Compound Name	M	C	No.	Compound Name	M	C
**1 ^a^**	(4-Methoxyphenyl) (phenyl)diazene	5.20	4.36	**70 ^c^**	Acid Red 151		3.68
**2 ^b^**	4-(2-Phenyldiazen-1-yl)benzoic acid	3.67	3.68	**71 ^c^**	Sudan Red 7B (Solvent Red 19)		3.68
**3 ^b^**	Methyl Red	3.68	3.68	**72 ^c^**	Acid Red 73 (Crocein Scarlet 3B)		3.68
**4 ^b^**	Methyl Red (sodium salt)	3.79	3.68	**73 ^c^**	Chlorantine Fast Red 5B		3.68
**5 ^a^**	2-Amino-5-[(*E*)-2-(2-carboxyphenyl)diazen-1-yl]benzoic acid	4.58	4.36	**74 ^a^**	Acid Black 1 (Amido Black 10B)	4.33	4.38
**6 ^a^**	4-{2-[4-(Diethylamino)phenyl]diazen-1-yl}benzoic acid	4.77	4.36	**75 ^a^**	Acid Black 1 (Salt)	4.41	4.38
**7 ^b^**	4-[2-(4-Hydroxyphenyl)diazen-1-yl]benzoic acid	3.32	3.68	**76 ^c^**	Naphthalene Blue Black CS		4.38
**8 ^b^**	4-[(*E*)-2-(2,4-dihydroxy-phenyl)diazen-1-yl]benzoic acid	3.31	3.68	**77 ^b^**	Direct Black 32	3.89	3.68
**9 ^b^**	5-[2-(4-Carboxyphenyl)diazen-1-yl]-2-hydroxybenzoic acid	3.35	3.68	**78 ^c^**	Direct Black 22 (Pontamine Fast Black PGR)		3.68
**10 ^c^**	2-{2-[4-(Phenylamino)-phenyl]diazen-1-yl}benzoic acid		4.36	**79 ^c^**	Acid Blue 113		4.36
**11 ^c^**	4-{2-[4-(Phenylamino)-phenyl]diazen-1-yl}benzoic acid		4.36	**80 ^a^**	Sudan Black B (Solvent Black 3)	4.20	4.36
**12 ^a^**	Metanil Yellow (sodium salt)	4.17	4.36	**81 ^b^**	Bordo Direct AN	3.86	3.68
**13 ^c^**	4-{2-[4-(Phenylamino)-phenyl]diazen-1-yl}benzene-1-sulfonic acid		4.36	**82 ^a^**	Congo Red	4.49	4.36
**14 ^b^**	Methyl Orange (Orange III)	3.74	3.68	**83 ^a^**	Direct Black 38	4.26	4.36
**15 ^c^**	4-{2-[4-(Diethylamino)-phenyl]diazen-1-yl}benzene-1-sulfonic acid		4.36	**84 ^c^**	Direct Green 6		4.36
**16 ^c^**	4-[2-(4-Hydroxyphenyl)diazen-1-yl]benzene-1-sulfonic acid		3.68	**85 ^c^**	Direct Green 1		3.68
**17 ^c^**	Tropaeolin O		3.68	**86 ^c^**	Congo Corinth G		4.36
**18 ^c^**	2-Hydroxy-5-[2-(4-sulfophenyl)diazen-1-yl]benzoic acid		3.68	**87 ^a^**	Acid Red 97	4.11	4.36
**19 ^c^**	Fast Yellow AB		3.68	**88 ^c^**	Acid Yellow 42		4.36
**20 ^a^**	5-Methyl-2-[2-(4-nitrophenyl)-diazen-1-yl]phenol	4.11	4.36	**89 ^a^**	Direct Blue 53 (Evans Blue)	5.13	4.36
**21 ^a^**	3-[(*E*)-2-(2,4-dihydroxyphenyl)-diazen-1-yl]-4-hydroxybenzene-1-sulfonic acid	4.27	4.36	**90 ^a^**	Trypanblue (Direct Blue 14)	4.09	4.36
**22 ^c^**	Sudan Orange G		3.68	**91 ^b^**	Direct Blue 15 (Aizen Direct Sky Blue 5B)	3.69	3.68
**23 ^b^**	4-[2-(2-Hydroxynaphthalen-1-yl)diazen-1-yl]benzoic acid	3.61	3.68	**92 ^b^**	Direct Blue 1	3.79	3.68
**24 ^c^**	4-[2-(1-Hydroxynaphthalen-2-yl)diazen-1-yl]benzoic acid		3.68	**93 ^c^**	Direct Blue 151		3.68
**25 ^b^**	4-[2-(4-Carboxyphenyl)diazen-1-yl]-3-hydroxynaphthalene-2-carboxylic acid	3.45	3.68	**94 ^a^**	Fast Blue B	4.30	4.36
**26 ^a^**	4-[2-(1-Aminonaphthalen-2-yl)diazen-1-yl]benzoic acid	4.73	4.36	**95 ^a^**	Tetrazolium Blue Chloride	4.04	4.36
**27 ^c^**	4-[2-(2-Aminonaphthalen-1-yl)diazen-1-yl]benzoic acid		4.36	**96 ^b^**	Brilliant Yellow	3.84	3.68
**28 ^b^**	Acid Orange 7	3.17	3.68	**97 ^b^**	5-[(*E*)-2-(2,4-Dihydroxyphenyl)-diazen-1-yl]-2-[(*E*)-2-{4-[(E)-2-(2,4-dihydroxyphenyl)-diazen-1-yl]-2-sulfophenyl}ethenyl]-benzene-1-sulfonic acid	3.73	3.68
**29 ^c^**	4-[2-(1-Hydroxynaphthalen-2-yl)diazen-1-yl]benzene-1-sulfonic acid		3.68	**98 ^b^**	2-{2-[4-(2-{4-[2-(3-Carboxy-4-hydroxy-phenyl)diazen-1-yl]-2-sulfophenyl}ethenyl)-3-sulfophenyl]diazen-1-yl}-5-hydroxybenzoic acid	3.39	3.68
**30 ^c^**	3-Hydroxy-4-[2-(4-sulfophenyl)-diazen-1-yl]naphthalene-2-carboxylic acid		3.68	**99 ^c^**	Direct Yellow 12		3.68
**31 ^c^**	Direct Yellow 11		3.68	**100 ^a^**	5-[2-(2-Hydroxy-naphthalen-1-yl)diazen-1-yl]-2-(2-{4-[2-(2-hydroxynaphthalen-1-yl)diazen-1-yl]-2-sulfophenyl}ethenyl)-benzene-1-sulfonic acid	4.55	4.36
**32 ^c^**	Acid Violet R		4.36	**101 ^b^**	4-{2-[4-(2-{4-[2-(3-Carboxy-2-hydroxy-naphthalen-1-yl)diazen-1-yl]-2-sulfophenyl}-ethenyl)-3-sulfo-phenyl]diazen-1-yl}-3-hydroxynaphthalene-2-carboxylic acid	3.87	3.68
**33 ^c^**	Sudan I		3.68	**102 ^b^**	4-Amino-3-{2-[4-(2-{4-[2-(1-amino-4-sulfonaphthalen-2-yl)diazen-1-yl]-2-sulfophenyl}ethenyl)-3-sulfophenyl]diazen-1-yl}naphthalene-1-sulfonic acid	3.74	3.68
**34 ^c^**	4-(2-Phenyldiazen-1-yl)naphthalen-1-ol		3.68	**103 ^b^**	Fluorescent Brightener 28	3.81	3.68
**35 ^c^**	Solvent Orange 7 (Sudan II)		3.68	**104 ^a^**	2-Hydroxy-4-[(*E*)-2-(4-{4-[(*E*)-2-(2-hydroxy-phenyl)diazen-1-yl]-benzamido}phenyl)-diazen-1-yl]benzoic acid	4.52	4.36
**36 ^c^**	Acid Ponceau 2G		3.68	**105 ^b^**	5-[(*E*)-2-(4-{4-[(*E*)-2-{2,4-dihydroxy-5-[(*E*)-2-(2-hydroxy-5-sulfo-phenyl)diazen-1-yl]-phenyl}diazen-1-yl]-benzamido}phenyl)-diazen-1-yl]-2-hydroxybenzoic acid	3.64	3.68
**37 ^c^**	Oil Yellow AB		3.68	**106 ^b^**	5-[2-(4-{4-[2-(3-Carboxy-4-hydroxyphenyl)diazen-1-yl]benzamido}-phenyl)diazen-1-yl]-2-hydroxybenzoic acid	3.44	3.68
**38 ^a^**	Acid Red 26 (Ponceau Xilidine)	4.06	4.36	**107 ^b^**	5-{2-[4-({4-[2-(6-Amino-1-hydroxy-3-sulfonaphthalen-2-yl)diazen-1-yl]-phenyl}carbamoyl)-phenyl]diazen-1-yl}-2-hydroxybenzoic acid	3.66	3.68
**39 ^b^**	Acid Orange 10 (Orange G)	3.34	3.68	**108 ^b^**	5-{2-[4-({4-[2-(7-Amino-1-hydroxy-3-sulfonaphthalen-2-yl)diazen-1-yl]-phenyl}carbamoyl)-phenyl]diazen-1-yl}-2-hydroxybenzoic acid	3.67	3.68
**40 ^b^**	Thorin	3.46	3.68	**109 ^b^**	4-Amino-3-[2-(4-{4-[2-(1-amino-4-sulfonaphthalen-2-yl)-diazen-1-yl]-benzamido}phenyl)-diazen-1-yl]-naphthalene-1-sulfonic acid	3.78	3.68
**41 ^b^**	Acid Red 29 (Chromotrope 2R)	3.65	3.68	**110 ^b^**	3-Hydroxy-4-[2-(4-{4-[2-(2-hydroxy-3,6-disulfonaphthalen-1-yl)diazen-1-yl]-benzamido}phenyl)-diazen-1-yl]-naphthalene-2,7-disulfonic acid	3.71	3.68
**42 ^b^**	Acid Red 1 (Azophloxin)	3.66	3.68	**111 ^b^**	5-Amino-3-{2-[4-({4-[2-(7-amino-1-hydroxy-3-sulfonaphthalen-2-yl)diazen-1-yl]phenyl}-carbamoyl)phenyl]diazen-1-yl}-4-hydroxy-naphthalene-2,7-disulfonic acid	3.58	3.68
**43 ^b^**	Acid Red 35	3.63	3.68	**112 ^a^**	4-(3-Methyl-4-{2-[4-(4-{2-[5-methyl-3-oxo-2-(4-sulfophenyl)-2,3-dihydro-1H-pyrazol-4-yl]diazen-1-yl}-benzamido)phenyl]-diazen-1-yl}-5-oxo-2,5-dihydro-1*H*-pyrazol-1-yl)benzene-1-sulfonic acid	4,02	4.36
**44 ^c^**	5-Amino-4-hydroxy-3-[2-(4-nitrophenyl)diazen-1-yl]naphthalene-2,7-disulfonic acid		4.36	**113 ^b^**	5-[2-(4-{[4-(2-{8-Amino-1-hydroxy-7-[2-(4-nitrophenyl)diazen-1-yl]-3,6-disulfonaphthalen-2-yl}diazen-1-yl)-phenyl]carbamoyl}-phenyl)diazen-1-yl]-2-hydroxybenzoic acid	3.95	3.68
**45 ^c^**	5-Amino-4-hydroxy-3-[2-(2-hydroxy-5-nitrophenyl)diazen-1-yl]naphthalene-2,7-disulfonic acid		3.68	**114 ^b^**	5-[2-(4-{[4-(2-{8-Amino-7-[2-(2-carboxy-4-nitrophenyl)diazen-1-yl]-1-hydroxy-3,6-disulfonaphthalen-2-yl}diazen-1-yl)phenyl]-carbamoyl}phenyl)-diazen-1-yl]-2-hydroxybenzoic acid	3.89	3.68
**46 ^c^**	2-[2-(1-Amino-8-hydroxy-3,6-disulfonaphthalen-2-yl)diazen-1-yl]-5-nitrobenzoic acid		4.36	**115 ^b^**	5-{2-[4-({4-[2-(6-Amino-1-hydroxy-5-{2-[2-(1-hydroxy-ethenyl)-4-nitro-phenyl]diazen-1-yl}-3-sulfonaphthalen-2-yl)diazen-1-yl]phenyl}-carbamoyl)phenyl]-diazen-1-yl}-2-hydroxybenzoic acid	3.86	3.68
**47 ^c^**	2-[2-(2-Amino-5-hydroxy-7-sulfonaphthalen-1-yl)diazen-1-yl]-5-nitrobenzoic acid		4.36	**116 ^b^**	4-Amino-3-{2-[4-({4-[2-(2,4-diamino-phenyl)diazen-1-yl]phenyl}carbamoyl)-phenyl]diazen-1-yl}-5-hydroxy-6-(2-phenyl-diazen-1-yl)-naphthalene-2,7-disulfonic acid	3.78	3.68
**48 ^c^**	Acid Red 4		3.68	**117 ^b^**	2-Hydroxy-5-{2-[4-(4-{2-[4-hydroxy-3-(phenylcarbamoyl)phenyl]diazen-1-yl}-benzamido)phenyl]-diazen-1-yl}-N-phenylbenzamide	3.78	3.68
**49 ^c^**	Solvent Red 1		3.68	**118 ^b^**	7-Benzamido-4-hydroxy-3-{2-[4-(4-{2-[4-hydroxy-3-(phenyl-carbamoyl)phenyl]diazen-1-yl}benzamido)-phenyl]diazen-1-yl}naphthalene-2-sulfonic acid	3.65	3.68
**50 ^c^**	Pigment Orange 5		4.36	**119 ^a^**	5-{2-[4-({4-[2-(6-Benzamido-1-hydroxy-3-sulfonaphthalen-2-yl)diazen-1-yl]-phenyl}carbamoyl)phenyl]diazen-1-yl}-2-hydroxybenzoic acid	4.65	4.36
**51 ^c^**	Acid Violet 7(Pontacyl Carmine 6B)		4.36	**120 ^a^**	4-Amino-3-{2-[4-(4-{2-[4-hydroxy-3-(phenyl-carbamoyl)phenyl]diazen-1-yl}benzamido)phenyl]-diazen-1-yl}naphthalene-1-sulfonic acid	4.60	4.36
**52 ^c^**	Acid Brown 4		3.68	**121 ^a^**	6-Amino-4-hydroxy-3-{2-[4-(4-{2-[4-hydroxy-3-(phenyl-carbamoyl)phenyl]diazen-1-yl}benzamido)-phenyl]diazen-1-yl}naphthalene-2-sulfonic acid	4.50	4.36
**53 ^c^**	Solvent Orange 2		3.68	**122 ^a^**	5-Amino-4-hydroxy-3-{2-[4-(4-{2-[4-hydroxy-3-(phenyl-carbamoyl)phenyl]-diazen-1-yl}-benzamido)phenyl]-diazen-1-yl}-naphthalene-2,7-disulfonic acid	4.50	4.36
**54 ^c^**	Solvent Red 3		3.68	**123 ^c^**	6-Hydroxy-5-{2-[4-(4-{2-[4-hydroxy-3-(phenylcarbamoyl)phenyl]diazen-1-yl}-benzamido)phenyl]-diazen-1-yl}-naphthalene-2-sulfonic acid		4.36
**55**	Acid Yellow 23 (Tartrazine, Food Yellow 4)	NOTE		**124 ^c^**	7-Amino-4-hydroxy-3-{2-[4-(4-{2-[4-hydroxy-3-(phenylcarbamoyl)-phenyl]diazen-1-yl}-benzamido)phenyl]-diazen-1-yl}naphthalene-2-sulfonic acid		4.36
**56 ^b^**	Acid Red 88 (Fast Red AV)	3.31	3.68	**125 ^c^**	4-Amino-5-hydroxy-6-{2-[4-(4-{2-[4-hydroxy-3-(phenylcarbamoyl)-phenyl]diazen-1-yl}benzamido)phenyl]-diazen-1-yl}naphthalene-1,3-disulfonic acid		4.36
**57 ^b^**	Acid Red 27 (Amaranth, Food Red 9)	2.33	3.68	**126 ^c^**	4,5-Dihydroxy-3-{2-[4-(4-{2-[4-hydroxy-3-(phenylcarbamoyl)-phenyl]diazen-1-yl}-benzamido)phenyl]-diazen-1-yl}-naphthalene-2,7-disulfonic acid		4.36
**58 ^a^**	Eriochrom Black T	4.13	4.36	**127 ^a^**	3-Hydroxy-4-{2-[4-(4-{2-[4-hydroxy-3-(phenylcarbamoyl)phenyl]diazen-1-yl}-benzamido)phenyl]-diazen-1-yl}-naphthalene-2,7-disulfonic acid	4.23	4.36
**59 ^c^**	Crystal Ponceau 6R		3.68	**128 ^a^**	2-Hydroxy-5-{2-[4-({4-[2-(2-hydroxynaphthalen-1-yl)diazen-1-yl]-phenyl}carbamoyl)-phenyl]diazen-1-yl}-N-phenylbenzamide	4.27	4.36
**60 ^b^**	Bismark Brown Y (Vesuvin)	3.87	3.68	**129 ^b^**	Direct Brilliant Orange	3.65	3.68
**61 ^c^**	Basic Brown 41		3.68	**130 ^c^**	Direct Red 23 (sodium salt)		3.68
**62 ^c^**	Basic Brown 4 (Bismark Brown R)		3.68	**131 ^c^**	Direct Orange 26		3.68
**63 ^b^**	Sudan IV (Scarlet Red)	3.05	3.68	**132 ^b^**	Direct Red 80 (Saturn Rot F3B)	3.80	3.68
**64 ^b^**	Solvent Red 25 (Sudan Red B)	3.42	3.68	**133 ^c^**	Direct Red 75		3.66
**65 ^b^**	Solvent Red 23 (Sudan III, Sudan V)	3.64	3.68	**134 ^c^**	Direct Yellow 50		4.36
**66 ^c^**	Solvent Red 26		3.68	**135 ^a^**	Fast Blue RR Salt	4.09	4.36
**67 ^c^**	Solvent Red 27 (Oil Red O)		3.68	**136 ^a^**	Fast Blue BB Salt	4.17	4.36
**68 ^b^**	Acid Red 66 (Biebrischer Scharlach)	3.95	3.68	**137 ^a^**	Fast Red B Salt	5.25	4.36
**69 ^b^**	Ponceau S	3.97	3.68	**138 ^c^**	Fast Black K Salt		4.36

Compound structures are presented in the Supplementary Material; ^a^ – “up”; ^b^ – “down”; ^c^ – “prediction”; NOTE – no observable toxic effect.

Considering that toxicity is constant in a range of ±0.50 log units (Köln model), the measured toxicity values Mlog(1/MRC_50_) are compared to the calculated average value Clog(1/MRC_50_). The introduction of this parameter points out the “class isotoxicity” character of the tested derivatives, which is also noticed in the case of AS-naphthols [9].

Depending on the experimental values, one could consider two calculated average values, and consequently an isotoxicity situated on two levels: Clog(1/MRC_50_) = 3.68 for “down” substrate (S) - receptor (R) interactions and 4.36 for “up” interactions (Table 1).

Just as in case of the Naphthol-AS [9], the measured toxicity values, F(1+2+…)e could be higher than the individual values of the reaction products F(1), F(2) *etc*., especially in the case of “strong” interactions. However, in this situation, one could not notice the additivity F(1+2+…)e > F(1+2+...)t (e = experimental; t = theoretical), as suggested by Backhaus [18] (Table 2). No synergistic effects were noticed.

For all the tested compounds, SUAD-subadditive toxicity values [9], e.g., (F(1 + 2+..)e < F(1 + 2+..)t, were found equal to zero. It is relevant to highlight the fact that none of the studied cases presented synergism, considering the individual toxicities of the metabolites. The lack of synergism is probably due to the metabolites’ reduced concentrations as compared to the initial xenobiotic, even if the individual toxicity was very high. For instance: 2,4-DHA: 2,4-dihydroxy aniline with M = 6.26.

The results obtained so far indicate that the dye (xenobiotic of first generation) is able to establish the reaction mechanism and the major antagonic effect through its chromophore. This effect is not influenced by the number, concentration, or the individual effectivenesses of the reaction products as simple functions (metabolites or second generation of xenobiotics).

**Table 2:** *Hydractinia echinata* test system: experimental (F(1+2+…)e), theoretical (F(1+2+...)t) and individual (F(1), F(2),…) toxicities of some azo- and azo-anilide derivatives and their products of enzymatic reduction and hydrolysis reaction.

No.	F(1+2+...)e	F(1)	F(1)	F(2)	F(2)	F(3)	F(3)	F(4)	F(4)	F(1+2+...)t
1	5.20	A	2.93 *	*p*-Methoxy-aniline	3.32	-	-	-	-	6.21
2	3.67	A	2.93 *	APAB	3.34	-	-	-	-	6.23
7	3.32	APAB	3.34	p-AF	5.78 *	-	-	-	-	9.12
8	3.31	APAB	3.34	2,4-DHA	6.26	-	-	-	-	9.60
9	3.35	APAB	3.34	Ac.p-AS	2.97	-	-	-	-	6.31
14	3.74	APABS	2.81	p-DMAA	3.50 *	-	-	-	-	6.31
20	4.11	p-NA	2.85	2-Aminocresol	3.21	-	-	-	-	6.06
96	3.84	ADASDS	3.43	p-AF( 2M)	5.78 *	-	-	-	-	14.99
97	3.73	ADASDS	3.43	2,4-DHA (2M)	6.26	-	-	-	-	15.95
104	4.52	Ac p-AmS	2.97	APAB	3.34	2,4-DHA	6.26	p-FDA	2.93 *	15.50
106	3.44	Ac p-AmS (2M)	2.97	APAB	3.34			p-FDA	2.93 *	9.24
117	3.78	Ac p-AmS (2M)	2.97	A (2M)	2.93 *	APAB	3.34	p-FDA	2.93 *	18.07

F(1), F(2),.. *etc*.: individual log(1/MRC_50_) toxicity values; SUAD-subadditive toxicity values (for which F(1 + 2+..)e < F(1 + 2+..)t); e = experimental; t = theoretical; A: aniline; APAB: *p*-aminobenzoic acid; *p*-NA: *p*-nitroaniline; ADASDS: diaminostilbene disulfonic acid; 4,4'-DABA: 4,4'-diaminobenzanilide; APABS: *p*-aminobenzenesulfonic acid; Ac.p-AmS: *p*-aminosalicylic acid; p-DMAA: *p*-dimethylaminoaniline; p-AF: *p*-aminophenol; 2,4-DHA: 2,4-dihydroxyaniline; p-FDA : *p*-phenylendiamine; molar concentration (2M) - in parenthesis; * Calculated values.

This idea is supported by: (a) the effectiveness of compounds 74 and 75, which are identical even if one contains the cyclohexyl-ammonium salt (simple function) as first generation xenobiotic. The ssme phenomenon was observed in case of derivatives 135 and 136; (b) the effectiveness of acetylene alcohols, which is determined by the presence of a reactive carbonyl group in the structure of intermediate metabolites and not by the hydroxy group included in the initial xenobiotic [19].

The energetically most stable structures are those in which an intramolecular hydrogen bond is formed between the hydroxyl, aminic hydrogen and nitrogen azo atoms, respectively, as derived from the restricted Hartree-Fock calculations. Usually the moiety including the azo group and the attached phenyl rings to this group are coplanar, except compounds 23, 25, 30, 31, 33, 35, 36, 38, 40, 41, 42, 43, 44, 45, 49, 50, 53, 56, 57, 59, 63, 64, 65, 66, 67, 68, 69, 70, 72, 101, 110, 123, 127, 128 in which the azo group is attached at position 1 to the naphthyl fragment, including a hydroxyl group in position 2 or 8, or a bulky (carboxylic, sulfonic acid, *etc*.) group is present in the *ortho* phenyl (naphthyl fragment) position with respect to the azo group e.g.,: 46, 47, 48, 51, 58, 71, 73, 74, 75, 77, 83, 84, 85, 107, 108, 111, 113, 114, 115, 116, 118, 119, 121, 122, 124, 126, 130, 131, 132, 133, 136, 137) where slight deviations from planarity are noted.

According to the tested derivatives’ structures, the following main reactions are possible: the azo group’s reductive cleavage, as well as the anilidic and ureic group’s hydrolysis. In biological systems which contain azo-reductases, the azo group can be easily reduced with formation of the corresponding amines [20].

In the case of the anilide group, its hydrolysis involves the formation of an oxyanionic intermediate during the rate determining step [9]. The electronic shift in the carbonyl oxygen’s direction, increases the C=O bond length from 1.2 Å (fundamental state) to 1.4 Å (transition state). The rehybridization of the carbonyl carbon from a planar sp^2^ structure to the tetrahedral sp^3^ structure, is associated with a translation movement of about 1.2 Å as compared to the double C=O bond, the formation of the oxyanionic structure, as well as with the intervention of the nucleophilic Ser195 agent [8]. The transition stage is stabilized by the presence of a water molecule [21], which is further used in the hydrolysis process.

For ureic groups’ hydrolysis, the molecule is separated into two parts, which can further react independently [10].

The effectiveness is not decisively influenced by the intramolecular H-bonds formation, but is dependent on the substituents’ and chromophores’ reciprocal positions, as well as by the molecule’s electronic delocalization possibilities. Thus, the presence of the hydroxyl group in the *ortho* position of the azo group, induces the simple quinoidic tautomery ST_OH_, through formation of a hydrazo derivative intermediate [22]: the hydrogen bond links the OH group to the non-adjacent nitrogen atom of the aromatic nucleus on which it is located.

According to the interatomic distance determinations ST_OH_ (* means partially) appears in derivatives 41, 42, 43, 58, 77*, 91*, 107, 111, 115, 116*, 118, 119, 121, 122 and 132* respectively, seems “unfavourable” in the presence of SO_3_H–azo H-bonds (39, 68), respectively OH–SO_3_H (69, 127), SO_3_H–SO_3_H (97, 100), OH–COOH (25, 101*), and do not “appear” in case of compound 128 (OH-NH). One notices some other cases: the “hydrogen in equilibrium” among OH–azo–OH (58) or OH–azo–SO_3_H bonds (91, 92, 132*), the simultaneous involvement of OH–SO_3_H in the H-bonds at the same azo group (107), as well as the formation of non-tautomeric H-bonds for derivatives 89, 91, 92, 110* and 132* (hydrogen bonds are presented in Table S3, Supplementary Information).

Gregory [20] has pointed out the presence of the *ortho* azo-aminoidic ST_NH2_, but due to its instability, the amino derivative remains as the azo form. In the present work, this matter of fact is specific for derivative 26, and probably for 83. Non-tautomeric H-bonds are noticed in the case of derivatives 60, 77*, 81, 82, 83*, 102, 109, 113, 114, 115*, 116 and 120, respectively.

The formation of adjacent cycles through multiple tautomeric-type H-bonds (in which both hydrogen atoms of the amino group are involved) of azo–OH–NH_2_–azo (74, 75, 83, 90, 111, 113, 114, 116) or azo–OH–NH_2_–SO_3_H (83, 89) is also possible.

The presence of two hydroxy or amino groups located on different aromatic nuclei separated by an azo group, or present on the same nucleus, but located between two azo groups (e.g., H acid), determines electronic shifts of the “push-pull” type and the double alternate tautomery (DAT). This fact is clearly specific in case of compounds 21 and 87. Due to the fact that the OH– group is more reactive than the NH_2_, one could also imagine “push-pull” type electronic shift in case of structures 74, 75 and 83*, respectively, and probably in the case of 113 and 116*.

The predicted effectiveness of 58 azo dyes were estimated taking into account the structural similarities of these compounds with the tested derivatives.

### 2.1. The “Down”Substrat-Receptor Interactions

One could notice that the absence of internal H-bonds in compounds 2–4, 7, 14 and, the presence of an internal H-bond between –OH and –COOH of derivatives 9, 25, 101, 105, 106, 117, the singular ST_OH_ of 8, 23, 28 and 108, the involvement of -SO_3_H group in ST_OH_ of 107 and 118 or in ST_NH2_ of 116, the non tautomeric amino-azo bonds of 81, 102, 109, 113–116, the steric hindrances of sulfonic groups at the double stilbenic bond level for 96–98, respectively, could generally contribute to some slightly reduced Mlog(1/MRC_50_) values.

The test system points out the steric effects, even if related to methyl substituents, which are less bulky [1]. This fact is more obvious in the case of Sudan IV (63), which has two methyl groups in the *ortho* position as compared to Sudan Red B (64) (with one methyl group), and Sudan III (65) (which does not have any methyl group and thus has the greatest effectiveness). This “sensibility” of the test-system was also noticed from the toxicity values of some nonyl-phenolic derivatives [23], but was not observed in case of *Daphnia magna* and duckweed test systems [16].

The effectiveness of derivatives of Sudan type can be correlated even with logP: the low reactivity of Sudan IV is due to its high lipophilicity (Table S2, Supplementary Information).

The steric hindrances corresponding to the dianisidinic’ –OCH_3_ group are stronger than those corresponding to the benzidinic’ –CH_3_ group, and this is why derivatives 91 and 92, with multiple H-bonds, involving even the methoxy group, exhibit lower toxicities than 89 and 90.

The identical inductive effects, but of opposite +M sign, exerted by two amino groups which can form non-tautomeric H-bonds, are responsible for the “down” interaction of derivative 60. It seems that the replacement of one –OH group by –NHCOCH_3_ in 41, or in 42 and 43, does not lead to modifications of their effectiveness.

The Amaranth molecule (57) exhibits a special polarity [24], and thus its lipophilic/hydrophilic balance (Table S2, Supplementary Information) is preponderantly in favor of the hydrophilic property. Therefore, the effectiveness of compound 56 which does not possess any sulfonic group is higher by 1 log unit.

According to the experimental values, the presence of the –SO_3_H group located in the *ortho* position to an azo group increases the effectiveness values close to “up” of derivatives 68 and 69, both by a negative mesomeric (–M) effect, as well as by affecting the molecules’ coplanarity.

In the case of azo-anilidic derivatives the general reaction mechanism involves the strong electron attracting effect of –M type of the carbonyl group, and the formation of a hydrazo derivative. This reduction is probably preceded by the anilide hydrolysis, since the carbonyl group is the permanently active reaction partner for Ser-195, and hence the reason of the electronic lack of balance. Moreover, one could emphasize that the anilide hydrolysis is faster than the azo group’s reduction, due to the fact that under identical experimental conditions, the total average effectiveness of naphthols-AS compounds is about 4.54 [9] as compared to the azo derivative’s value, which is equal to 4.01.

Because an anilide group can activate a single azo group, the anilide hydrolysis and the reduction of the intermediate hydrazo derivative are processes which occur fast. The reaction rate-determining step is represented by the reduction of the second and the third azo group. This is the case of derivatives 105–111 and 113–116, even if 113–115, also possess NO_2_ substituents. In the case of derivatives 117 and 118, except for the internal H-bonds, the three anilide groups exhibit antagonistic electronic effects, the azo group’s activation takes place successively, and the reaction rate is also lower. Thorin I (40), can be included in the present series of Mlog(1/MRC_50_) values, and this fact leads to the conclusion that the arsenic and the sulfonic groups are equivalent.

### 2.2. The “Up”Substrate-Receptor Interactions

The effectivenesses of the monoazo, dis- and polyazo derivatives are dependent on different combinations of stereo-electronic effects: a direct action of the *ortho* or *para* –COOH group by –M (mesomeric) effect for compounds 5 and 26, lipophilic influences for 6, 12, 38, the –M effect of –NO_2_ in 20, 74 and 75, as well as the DAT effect in the case of 21 and 58. More energic electronic shifts can also appear when H-bonds are involved in the formation of adjacent cycles, where atoms exhibit different electronegativities, e.g., in: 74, 75, 89, 90, 119 and 122.

Congo Red (82) (characterized by a total symmetry), Direct Black 38 (83) (has four H-bonds) and 100 (characterized by DAT) have very close effectiveness values.

The distinguished effectiveness of 89* can be explained by its total symmetry and DAT. In case of 87 and 90 derivatives, the steric hindrances caused by the –SO_3_H groups located in the vicinity of the azo groups yield the lowering of the effectiveness by 1 log unit.

Except compound 104 whose azo:anilidic group ratio is 2:1, in the case of derivatives 119–122, 127 and 128, this ratio is equal to 2:2. In this situation, two anilide groups (e.g., in salicylanilide and diaminobenzanilide) are each able to activate an azo group, and thus the hydrolysis and the reduction processes occur fast, and the compounds’ toxicity is higher. As compared to compound 104 who’s carbonylic –M effect is intensified by its two hydroxy resorcinol groups, in the case of derivative 105 their influence is diminished by the coupling with 2-aminophenol-4-sulfonamide.

4-Methoxyazobenzene has a coplanar structure and, considering the influence of the positive mesomeric (+M) effect of the –OCH_3_ group, an extended conjugation favours the formation of the corresponding hydrazo derivative. Though the *para* alkoxy derivatives exhibit carcinogenic effects [25], the introduction of alkoxy groups in the *ortho* position of aminoazobenzene dyes decreases their mutagenic effect [26].

### 2.3. Azo-Heterocyclic Derivatives and Diazonium Salts

The presence of some heterocyclic components can influence differently the effectiveness. In a first analysis, the increased effectiveness exhibited by Sudan Black B (80) and the compound 112 could be assigned to their 2,3-dihydro-1*H*-perimidine (80), or pyrazolone rings (112), and in the case of derivatives which do not possess azo groups such as Tetrazolium Blue Chloride (95) and Fluorescent Brightener (103), to the combinations of tetrazolium-*ortho*-dianisidine type, as well as of triazinic-diaminostilbene-disulfonic acid type. The diazonium salts such as Fast Blue B (94), Fast Blue RR Salt (135) and Fast Blue BB Salt (136), can be characterized by a stronger electrophilic character, and this fact is probably due to the –M effect of the nitro group in case of the derivative Fast Red B Salt (137). The pyrazole-monoazo dye Tartrazine (55) is not toxic at all, even at a concentration of 0.11 mol/L, in accordance to its increased hydrophilicity.

## 3. Experimental Section

### 3.1. Test Substances

The test conditions and method were identical to those described in a previous work [9]. Some of the test substances (Table 1) were purchased from catalogues, or were synthesized at the Institute of Chemistry of Timisoara of the Romanian Academy, Romania (derivatives No.: 1, 2–4, 7–9, 12, 20, 21, 23, 25, 26, 77, 87, 96–98, 100–102, 104–122, 127–129, 132).

### 3.2. Test Organism

Colonies of *H. echinata* (Biologische Anstalt, Helgoland, Germany) were used to obtain eggs and larvae. The culture medium was artificial seawater (980 mosmol, pH 8.2, 18 °C). In laboratory an artificial metamorphosis can be synchronically started by the introduction of Cs^+^ ions or by using seawater without Mg^2+^ ions; it then lasts only 24 h. Under the action of external stimulus of Cs^+^ ions or Cs^+^ ions together with the tested compounds, one part of larvae further lives as such and another one is metamorphosized to the polyp form. The evaluation of the influence of the tested substance is very clear this way, the proposed method being based on this aspect.

### 3.3. Toxicity Test: Induction of Metamorphosis and Treatment with Test Substances

*H. echinata* larvae were exposed to seawater containing Cs^+^ and simultaneously one of the test substances for 3 h. The percentage of animals that underwent metamorphosis (development into polyps) was determined after 24 h. During the following days the frequency of inductions did not further increase. We chose a concentration of inducers which caused about three half to three quarters of the larvae to metamorphose in order to have conditions which are highly sensitive against an inhibitory influence. The concentration of the test substances (expressed in mol/L) was varied in such a way that we were able to determine the concentration at which the frequency of induction was reduced by 50% with respect to a control. This concentration was termed MRC_50_ (for *M*etamorphosis *R*eduction *C*oncentration) and is similar to the effective EC_50_ concentration that gives half maximal effective response [9,23].

### 3.4. Theory/Calculation

The neutral molecular structures of azo dyes (Table 1) were modeled by the conformational search ability of the Omega v.2.4.3 (OpenEye Scientific Software, Santa Fe, NM. USA) program [27,28,29] (only structures having toxic effect on *H. echinata* test system were considered). SMILES notation was used as program input.

The following parameters were used for the conformer generation with Omega: a maximum of 400 conformers per compound, an energy cutoff of 10 kcal/mol, relative to a global minimum, which was identified from the search. The force field used was the 94s variant of the Merck Molecular force field (MMFF) with Coulomb interactions and the attractive part of the van der Waals interactions. To avoid redundant conformers, any conformer having a RMSD fit outside the range between 0.1 and 0.5 Å to another conformer was removed.

The minimum energy conformers thus obtained for each dye compound were used as input structures and were fully geometry optimized at restricted Hartree-Fock (3-21G basis set) level of theory (a*b initio* data are presented in Table S1, Supplementary Material) using the Gaussian 2009 software [30]. All optimized structures were characterized as true minima by frequency calculations (NImag = 0 for each compound).

Dye hydrophobicity, solubility and other molecular properties (Table S2, Supplementary Material) were evaluated by several programs. Thus, the logarithm of the octanol-water partition (logP) coefficient was calculated by the InstantJChem 5.12.4 (2013, Chemaxon) software [31], as well as other molecular properties: average polarisability, the number of donor, respectively acceptor H-bonds. Instant JChem was used for structure database management, search and prediction. The solubility (logS) was predicted by the AlogPS 2.1 program [32].

## 4. Conclusions

In conclusion, this study demonstrates the possibility of toxicity determination of some azo dyes and diazonium salts using the *He*TS, which does not affect environmental equilibrium because the animals used in these experiments continue to live as larvae or polyps (as a result of metamorphosis). The included toxicity predictions illustrate the economy of time and research resources which could be done by adopting this procedure, in accordance to the “3Rs” concept as alternative method of investigation [33] or examination in advance, which leads to the replacement of other animal species in toxicity testing [34]. The test-system is characterized by reproducibility; it is fast, accessible, simple and offers a practical alternative in the research activity of different fields of interest, such as drugs or new classes of chemical derivatives. The main enzymatic reactions are the reduction of the azo group and the hydrolysis of the amido group. They are probably competitive and dependent on the limited or total coplanarity of the molecules, as well as by the existing electronic delocalization possibilities. Thus, it is possible that hydrolysis could occur prior to the reduction reaction, since the carbonyl anilide group is an active reaction center, as well as a permanent partner for Ser195. The S-R interaction, and therefore the effectiveness, are not dependent on the number of azo groups, but is influenced by the number of anilide groups. The toxicity is not dependent directly on the molecular dimension, as Protic and Sabljic [35] observed for commercial chemicals. The close Mlog/(1/MRC_50_) values lead to the determination of the average Clog(MRC_50_) value, which is characteristic for a class of compounds. One could emphasize the “class isotoxicity”. The reduction and the hydrolysis reaction products are mixtures of aromatic amines. The problem of the reaction mixture’s effectiveness and the influence of the methoxy substituents are not fully clarified. The calculated non-empiric molecular parameters could be used in different QSAR computations, especially for the determination of the molecules’ degree of penetration across the vascular endothelium into the brain (BBB). This issue could be one of the most important solutions in the research of new specific therapeutic agents [36].

The study does not involve clinical studies or patient data. Even if the biological differences between *H. echinata* and the superior organisms do not allow simple extrapolations, this test-system points out the toxicity trends at a cellular level. They represent more than surrogates for tests on superior organisms due to fact that it was discovered by experimental system with bacterium *E. coli* (Jacob and Monod, *The Nobel Prize in Physiology or Medicine*, 1965) that is fundamental to cellular regulation for all organisms, or: “what is valid for bacteria, is also valid for mammals” [37].

## Supplementary Materials

Supplementary materials can be accessed at: http://www.mdpi.com/1420-3049/19/7/9798/s1.
