# Supplementary material for: The Hydractinia echinata Test-System. III: Structure-Toxicity Relationship Study of Some Azo-, Azo-Anilide, and Diazonium Salt Derivatives
Source: Molecules. 2014 Jul 8;19(7):9798–817. doi: 10.3390/molecules19079798 (PMC6270994; doi:10.3390/molecules19079798)

Supplementary Information

Table S1. Structure of azo-, azo-anilide, diazonium salts derivatives and a*b initio* data for the neutral energy minimized molecules.

No	CAS	CI	Compound Structure	E(HF/ 3-21G) (a.u.)	ZPE (Hartree/Particle)	Hcorr	Gcorr
**1** [a]	2396-60-3		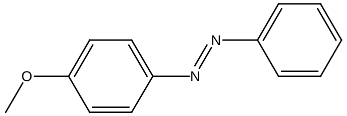	−679.16	0.24	0.26	0.20
**2 *** [b]			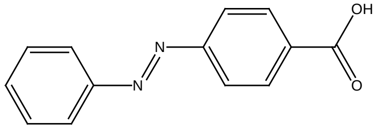	−752.48	0.22	0.24	0.18
**3** [b]	493-52-7	13020	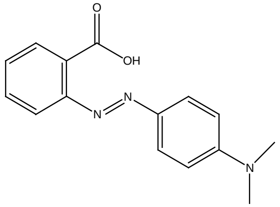	−807.20	0.30	0.32	0.25
**4 *** [b]	845-10-3	13020	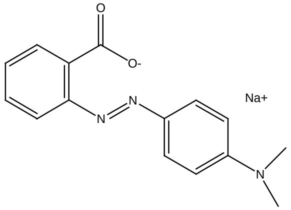	−806.61	0.23	0.24	0.18
**5 *** [a]			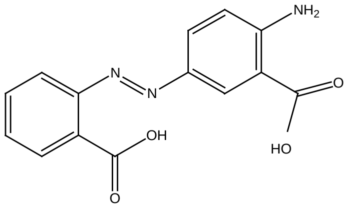	−993.77	0.26	0.27	0.21
**6** [a]			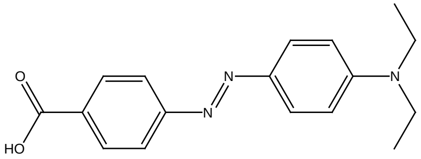	−962.47	0.36	0.38	0.31

**7*** [b]			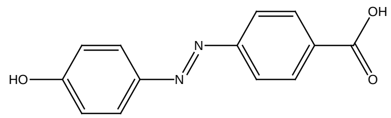	-826.92	0.23	0.24	0.18
**8 *** [b]			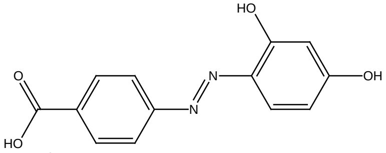	−901.36	0.23	0.25	0.19
**9** [b]			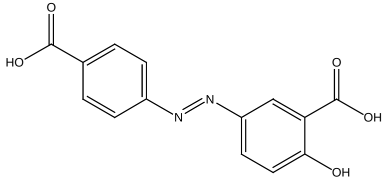	−1013.48	0.24	0.26	0.20
**10** [c]			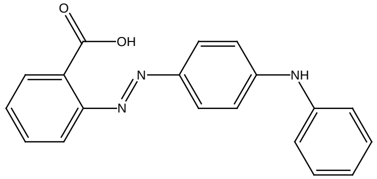	−1035.46	0.33	0.35	0.28
**11** [c]			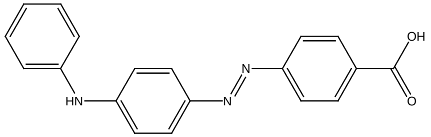	−1035.47	0.33	0.35	0.28
**12** [a]	587-98-4	13065	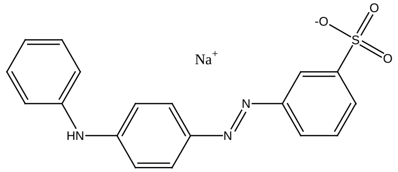	−1467.59	0.33	0.35	0.27
**13** [c]			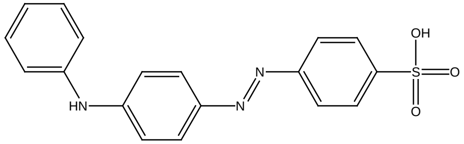	−1467.59	0.33	0.35	0.27
**14** [b]	547-58-0	13025	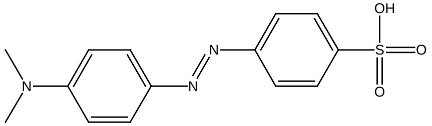	−1316.94	0.30	0.32	0.25

**15** [c]			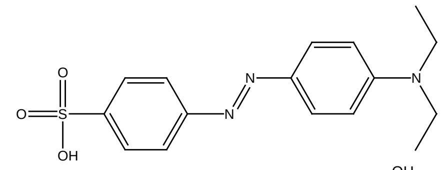	−1394.58	0.36	0.38	0.38
**16** [c]			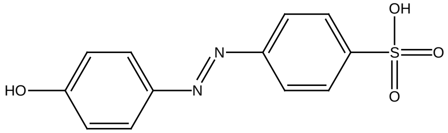	−1259.04	0.22	0.24	0.18
**17** [c]	547-57-9		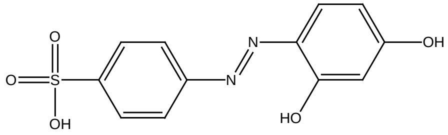	−1333.47	0.23	0.25	0.18
**18** [c]			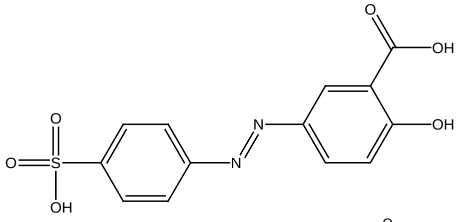	−1445.60	0.24	0.26	0.19
**19** [c]	2706-28-7	13015	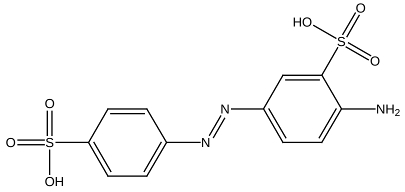	−1858.02	0.25	0.27	0.20
**20** [a]			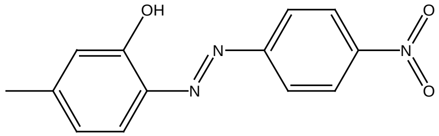	−881.46	0.24	0.26	0.20
**21** [a]			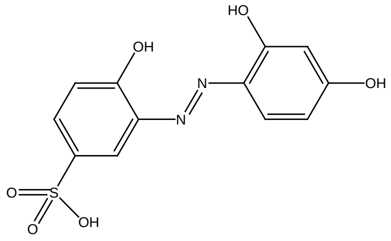	−1407.91	0.23	0.25	0.18

**22** [c]	2051-85-6	11920	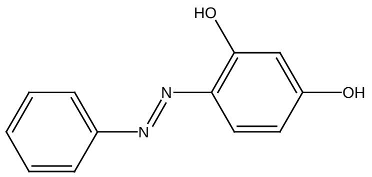	−714.79	0.22	0.23	0.18
**23** [b]			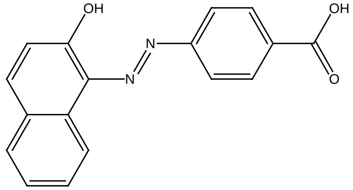	−978.70	0.28	0.28	0.23
**24** [c]			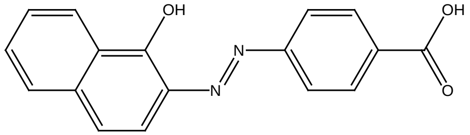	−978.73	0.28	0.30	0.23
**25** [b]			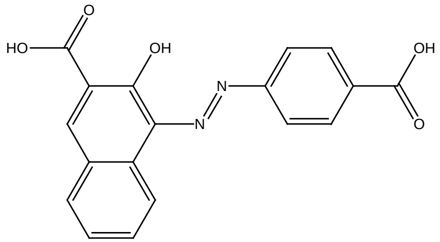	−1165.29	0.29	0.31	0.24
**26** [a]			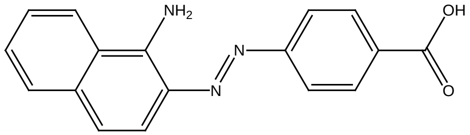	−959.00	0.29	0.31	0.25
**27** [c]			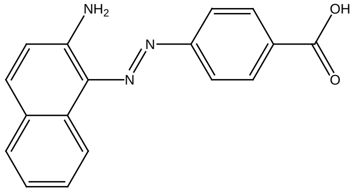	−959.01	0.29	0.31	0.25

**28** [b]	633-96-5	15510	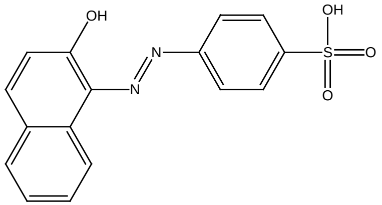	−1410.82	0.28	0.29	0.23
**29** [c]			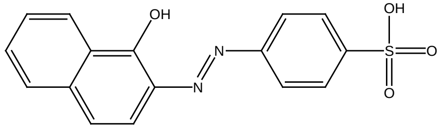	−1410.84	0.28	0.29	0.23
**30** [c]			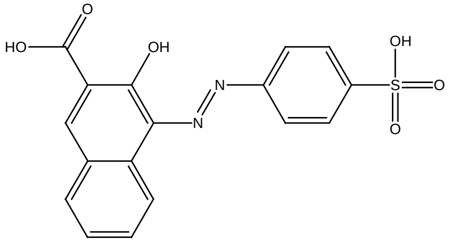	−1597.40	0.29	0.31	0.24
**31** [c]	1325-37-7	40000	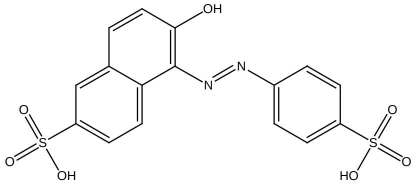	−2029.51	0.29	0.31	0.23
**32** [c]	6441-91-4	17025	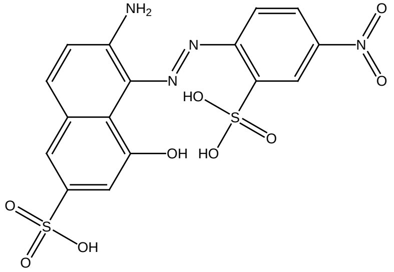	−2286.54	0.33	0.36	0.28
**33** [c]	842-07-9	12055	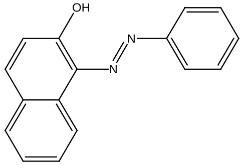	−792.13	0.26	0.28	0.22

**34** [c]			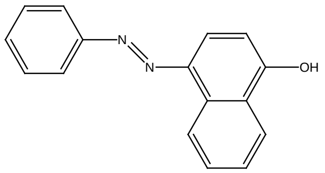	−792.15	0.26	0.28	0.22
**35** [c]	3118-97-6	12140	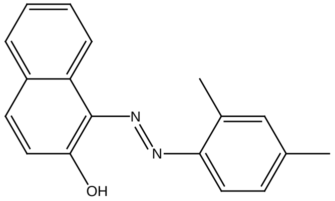	−869.78	0.32	0.32	0.27
**36** [c]	5859-00-7	16100	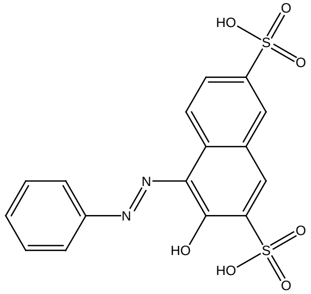	−2029.51	0.29	0.31	0.23
**37** [c]	85-84-7	11380	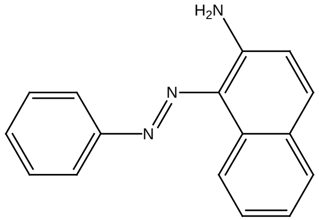	−772.44	0.28	0.29	0.24
**38** [a]	3761-53-3	16150	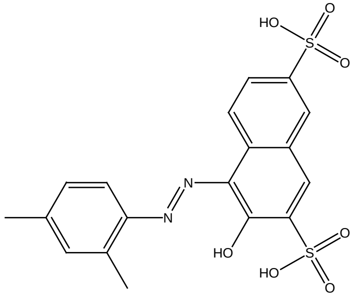	−2107.16	0.35	0.37	0.29

**39** [b]	1936-15-8	16230	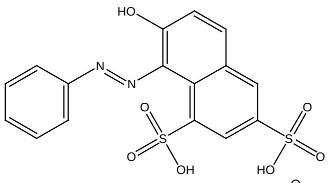	−2029.50	0.29	0.31	0.24
**40** [b]	3688-92-4		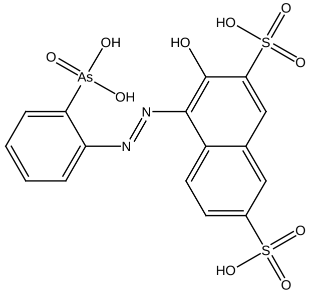	-	-	-	-
**41** [b]	4197073	16570	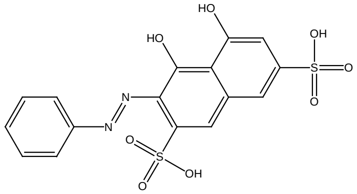	−2103.96	0.29	0.32	0.24
**42 *** [b]	3734-67-6	18050	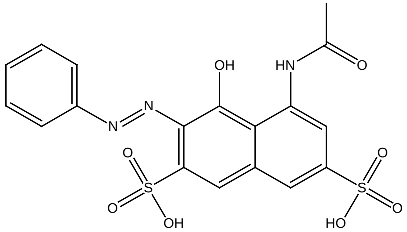	−2235.20	0.35	0.38	0.29
**43 *** [b]	6441-93-6	18065	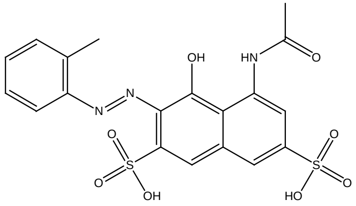	−2274.02	0.38	0.41	0.32

**44** [c]			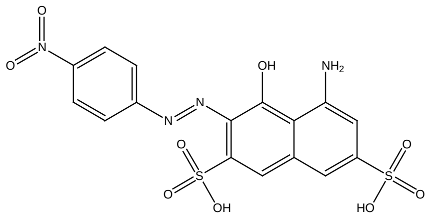	−2286.55	0.31	0.34	0.25
**45** [c]			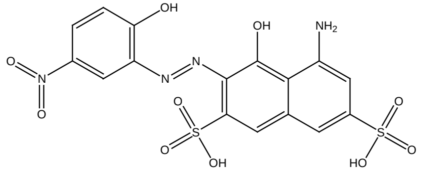	−2360.99	0.31	0.34	0.26
**46** [c]			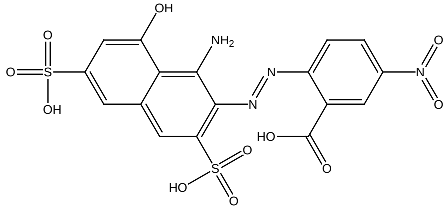	−2473.09	0.33	0.35	0.26
**47** [c]			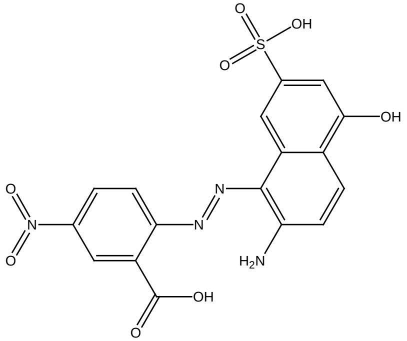	−1854.42	0.31	0.34	0.26
**48** [c]	5858-39-4	14070	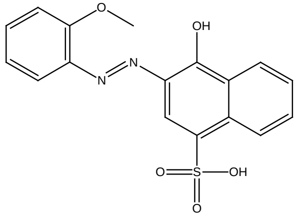	−1524.08	0.31	0.33	0.26

**49** [c]	1229-55-6	12150	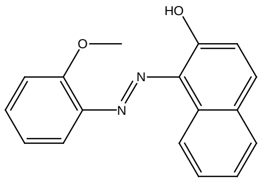	−905.39	0.30	0.31	0.25
**50** [c]	3468-63-1	12075	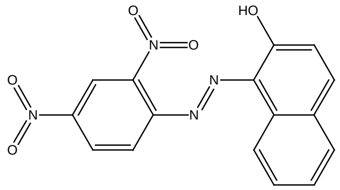	−1196.70	0.27	0.28	0.22
**51** [c]	4321-69-1	18055	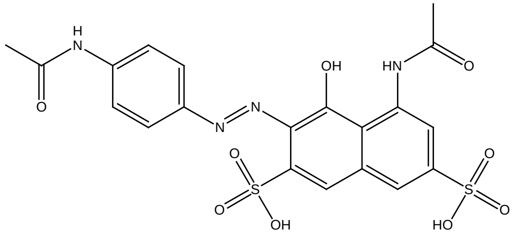	−2440.86	0.41	0.44	0.34
**52** [c]	5858-51-5	14805	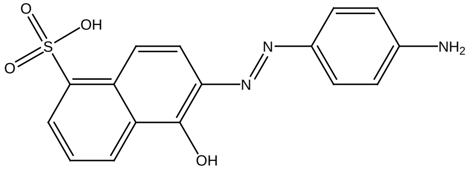	−1466.74	0.32	0.34	0.27
**53** [c]	2646-17-5	12100	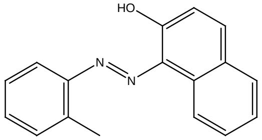	−830.96	0.29	0.31	0.25
**54** [c]	6365-42-8	12010	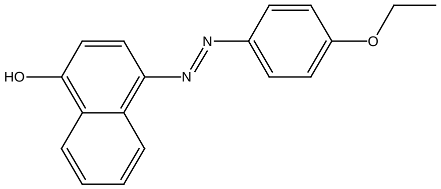	−944.23	0.33	0.35	0.28

**55 ***	1934-21-0	19140	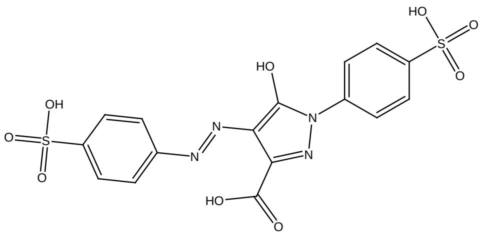	−2286.67	0.31	0.34	0.25
**56** [b]	1658-56-6	15620	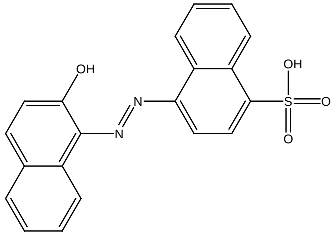	−1562.62	0.33	0.35	0.28
**57 *** [b]	915-67-3	16185	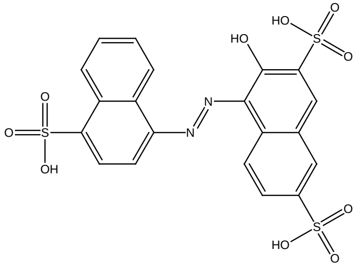	−2800.00	0.35	0.38	0.29
**58 *** [a]	1787-61-7	14645	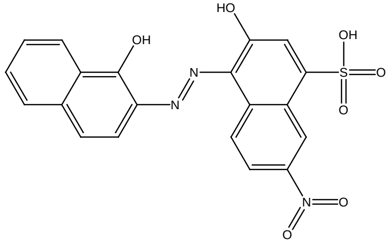	−1839.36	0.33	0.36	0.28
**59** [c]	2766-77-0	16250	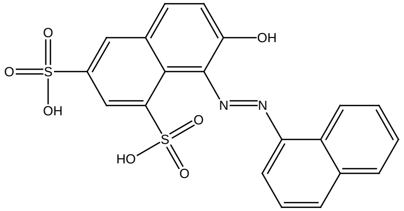	−2181.30	0.34	0.37	0.29

**60** [b]	1052-38-6	21000	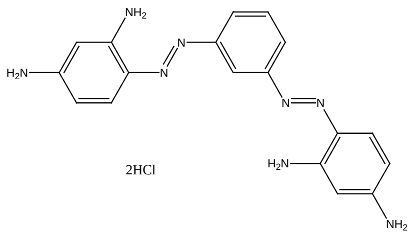	−1121.31	0.38	0.40	0.32
**61** [c]	1052-38-6	21000:1	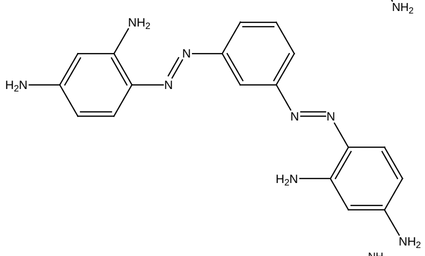	−1121.31	0.38	0.40	0.32
**62** [c]	8005-78-5	21010	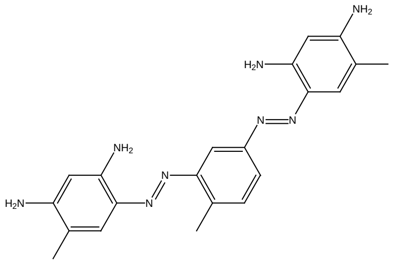	−1237.77	0.46	0.49	0.41
**63** [b]	85-83-6	26105	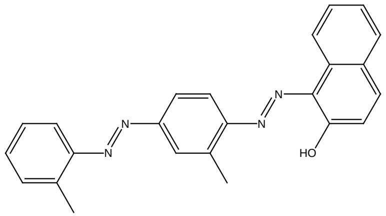	−1206.27	0.42	0.44	0.36
**64** [b]	3176-79-2	26110	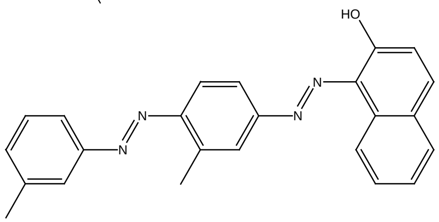	−1206.26	0.42	0.44	0.36

**65** [b]	85-86-9	26100	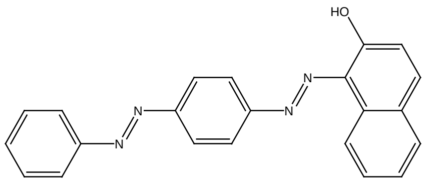	−1128.62	0.36	0.38	0.31
**66** [c]	4477-79-6	26120	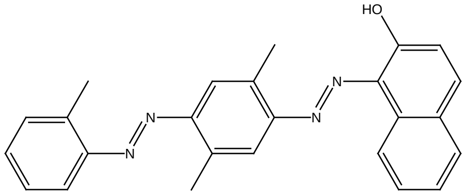	−1245.09	0.45	0.47	0.39
**67** [c]	1320-06-5	26125	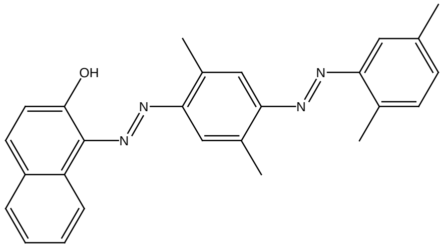	−1283.91	0.48	0.50	0.42
**68** [b]	4196-99-0	26905	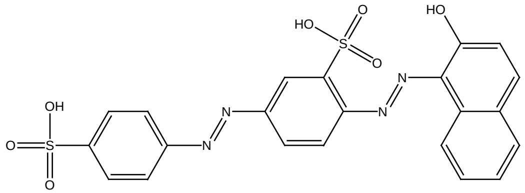	−2366.00	0.39	0.42	0.33
**69** [b]	6226-79-5	27195	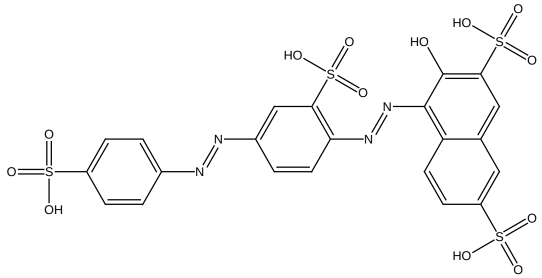	−3603.37	0.41	0.45	0.34

**70** [c]	6406-56-0	26900	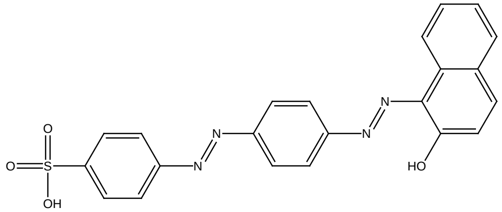	−1747.31	0.37	0.37	0.31
**71** [c]	6368-72-5	26050	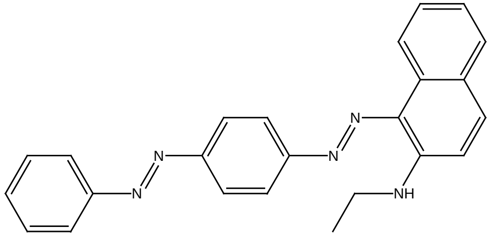	−1186.55	0.44	0.46	0.38
**72** [c]	5413-75-2	27290	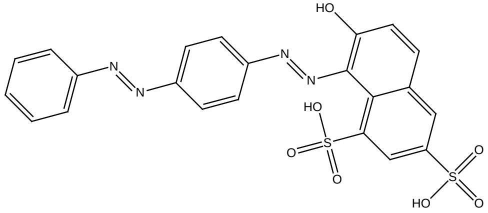	−2365.98	0.39	0.42	0.32
**73** [c]	259636	28160	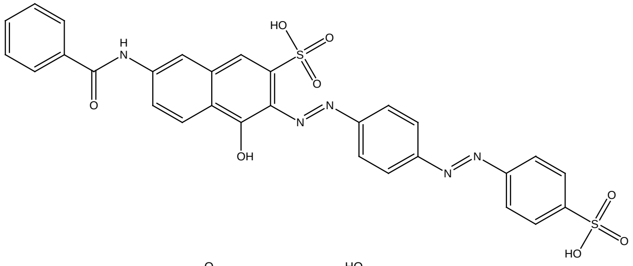	−2761.14	0.51	0.54	0.43
**74 *** [a]	1064-48-8	20470	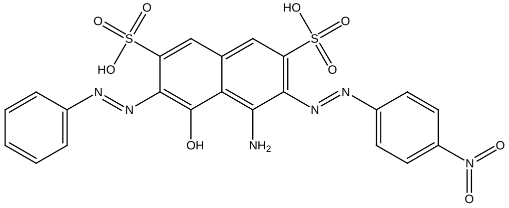	−2623.04	0.41	0.44	0.34

**75** [a]			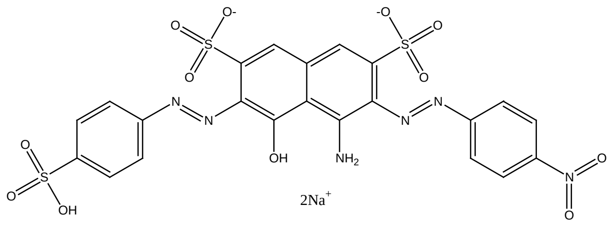	−3241.73	0.42	0.46	0.35
**76** [c]		20480	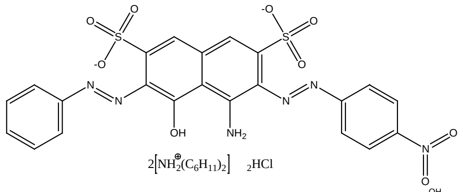	−2623.04	0.41	0.44	0.34
**77** [b]	6428-38-2	35440	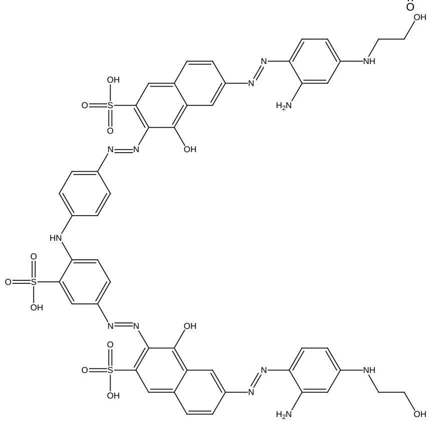	−4689.99	0.97	1.03	0.85

**78** [c]	6473-13-8	35435	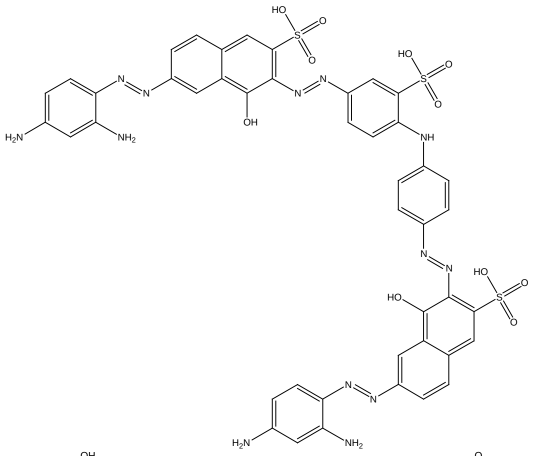	−4385.86	0.83	0.89	0.73
**79** [c]	530-08-9	26360	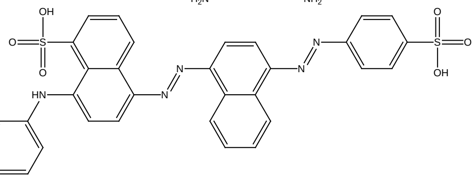	−2726.35	0.54	0.58	0.47
**80** [a]	4197-25-5	26150	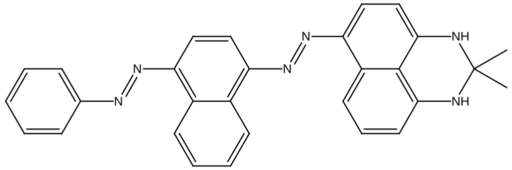	−1430.74	0.51	0.54	0.45
**81** [b]	2429-84-7	22150	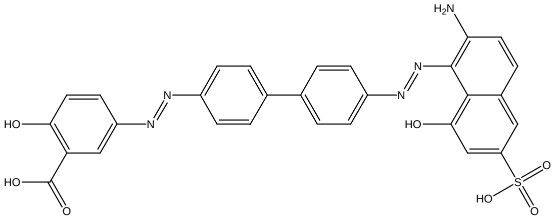	−2291.31	0.50	0.53	0.43

**82 *** [a]	573-58-0	22120	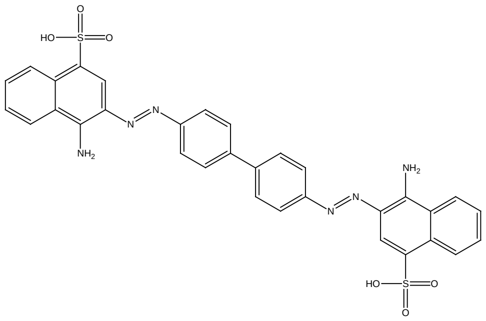	−2781.10	0.56	0.60	0.48
**83** [a]	1937-37-7	30235	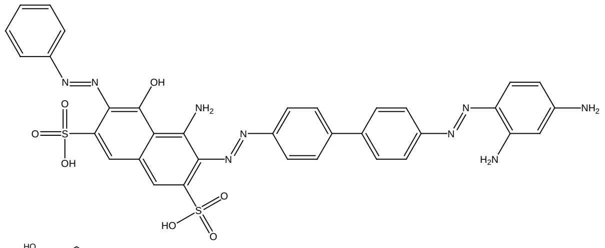	−3094.97	0.63	0.67	0.55
**84** [c]	4335095	30295	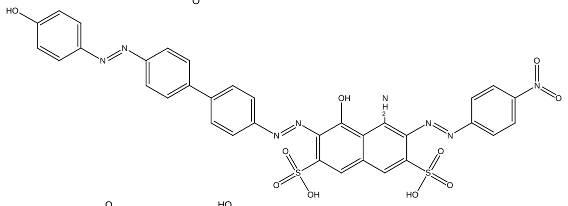	−3262.24	0.60	0.64	0.52
**85** [c]	3626-28-6	30280	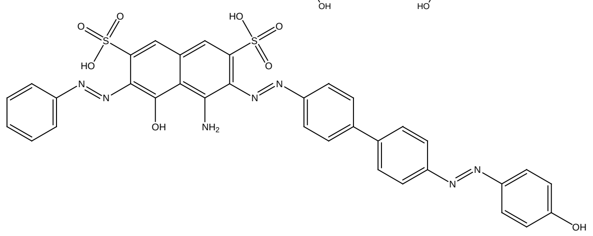	−3059.95	0.60	0.64	0.52

**86** [c]	2429-70-1	22145	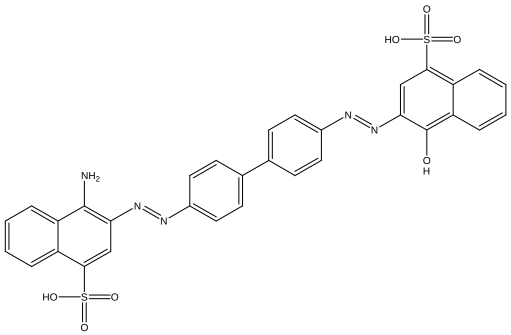	−2800.82	0.55	0.58	0.47
**87** [a]	10169-02-5	22890	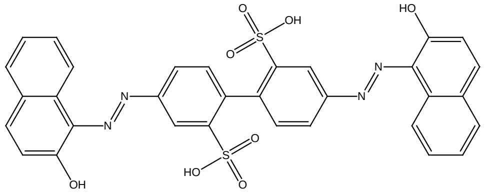	−2820.48	0.47	0.57	0.45
**88** [c]	6375-55-9	22910	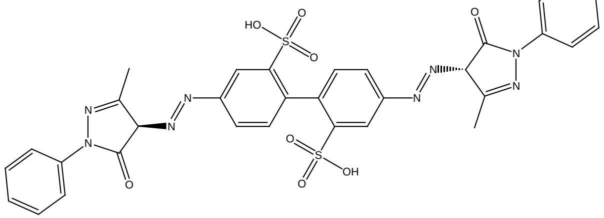	−3039.31	0.60	0.64	0.51
**89** [a]	314-13-6	23860	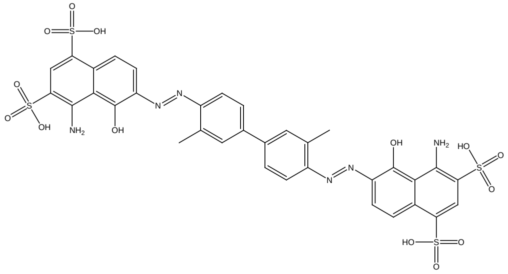	−4245.04	0.66	0.71	0.57

**90** [a]	72-57-1	23850	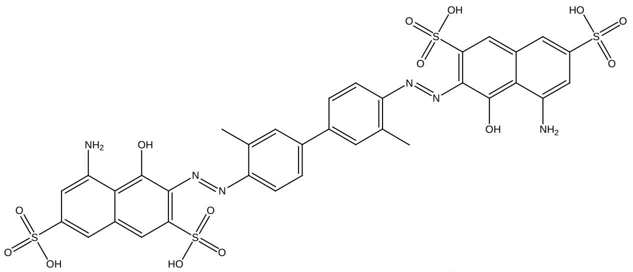	−4245.01	0.65	0.71	0.56
**91** [b]	2429-74-5	24400	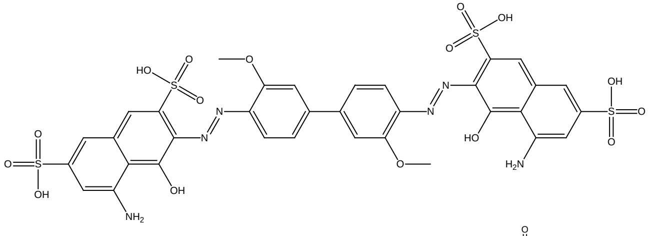	−4393.88	0.67	0.72	0.57
**92 *** [b]	2610051	24100	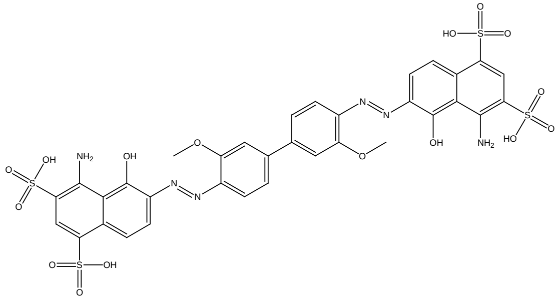	−4393.90	0.67	0.72	0.57
**93** [c]	110735-25-6	24175	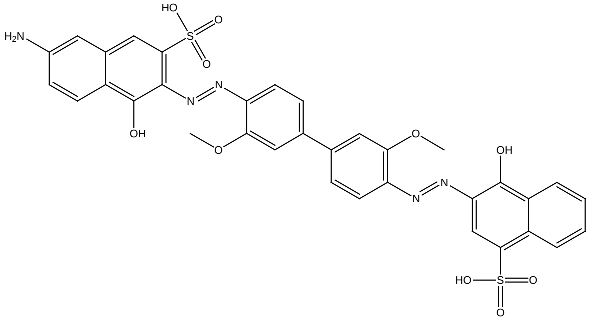	−3101.77	0.62	0.66	0.54
**94 *** [a]	14263-94-6		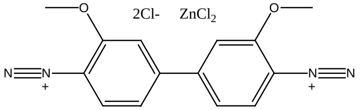	−899.27	0.54	0.27	0.21

**95** [a]	1871-22-3		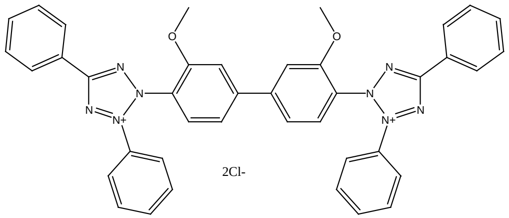	−2106.49	0.70	0.74	0.62
**96** [b]	3051114	24890	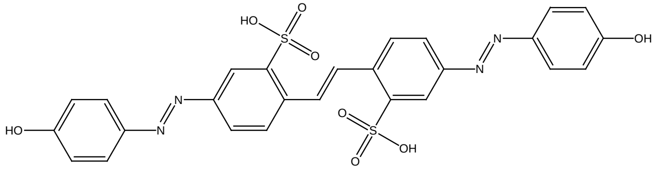	−2593.40	0.47	0.50	0.40
**97** [b]			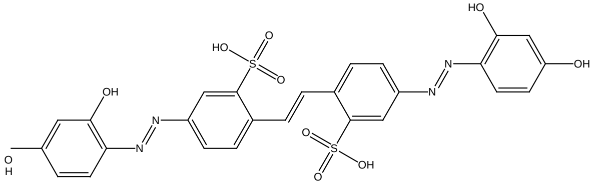	−2742.27	0.47	0.51	0.40
**98** [b]			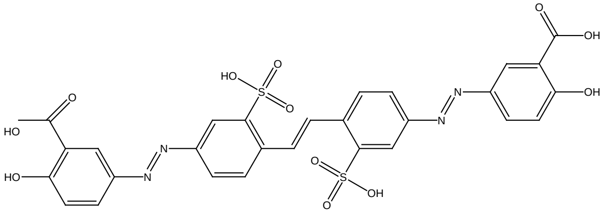	−2966.51	0.50	0.54	0.54
**99** [c]	2870-32-8		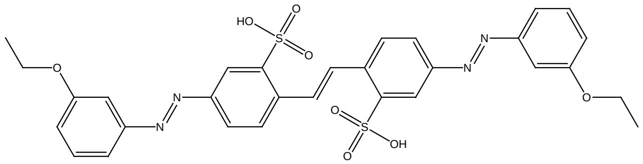	−2748.66	0.59	0.63	0.51
**100 *** [a]			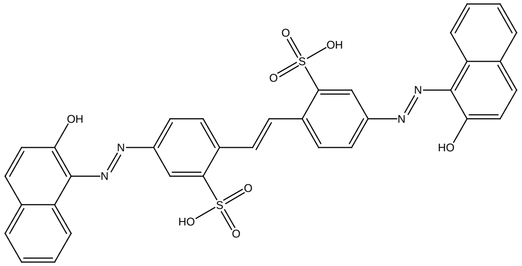	−2896.96	0.57	0.60	0.49

**101** [b]			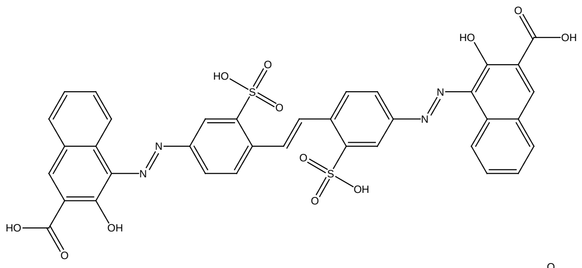	−3270.12	0.60	0.64	0.64
**102** [b]			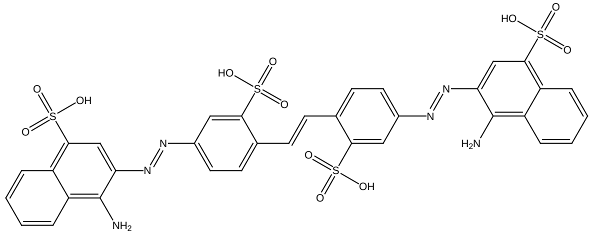	−4094.95	0.62	0.67	0.54
**103 *** [b]	4404-43-7	40622	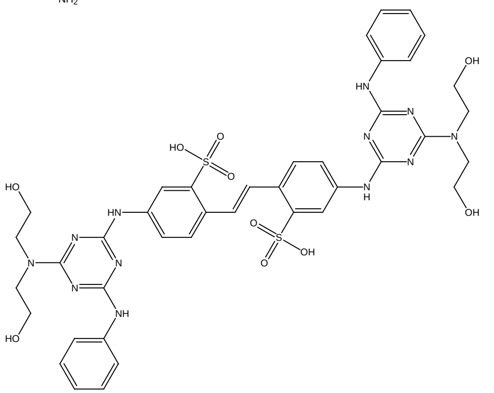	−3716.76	0.91	0.97	0.81
**104** [a]			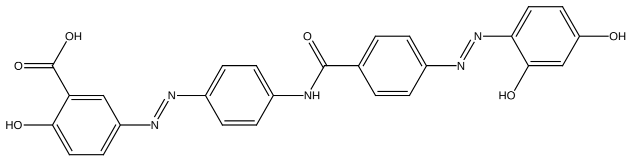	−1707.39	0.45	0.48	0.39

**105** [b]	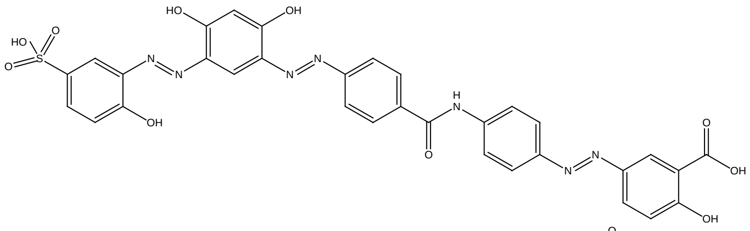	−2717.28	0.58	0.62	0.50
**106** [b]	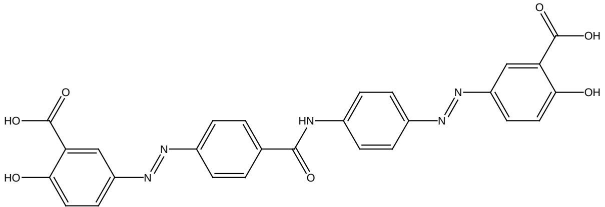	−1819.54	0.47	0.50	0.40
**107** [b]	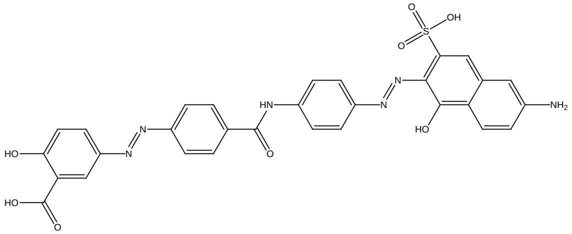	−2458.19	0.53	0.57	0.46
**108** [b]	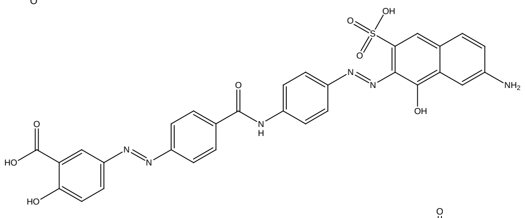	−2458.18	0.53	0.57	0.46
**109** [b]	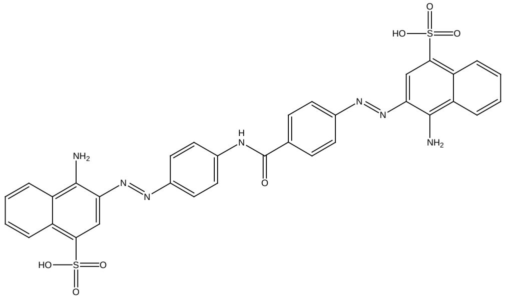	−2947.95	0.59	0.63	0.51

**110** [b]	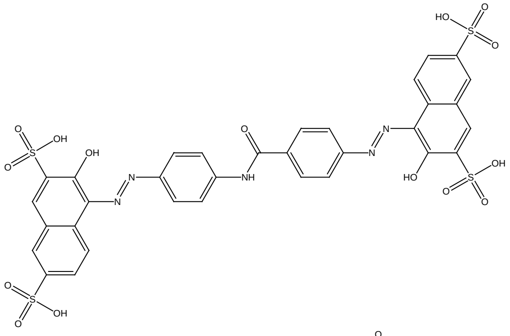	−4224.73	0.59	0.64	0.49
**111** [b]	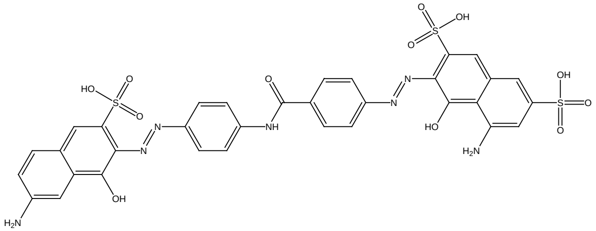	−3715.53	0.61	0.66	0.53
**112** [a]	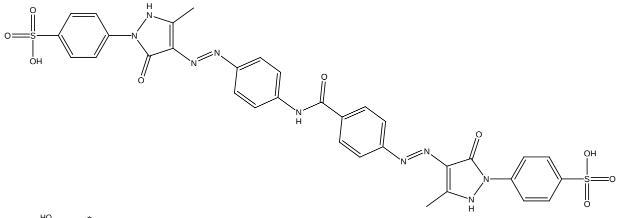	−3206.18	0.63	0.68	0.54
**113** [b]	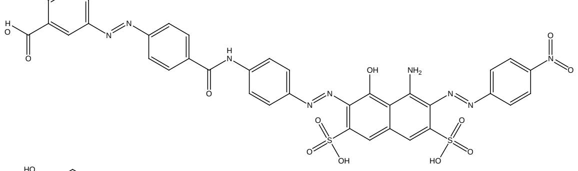	−3615.65	0.65	0.69	0.55
**114** [b]	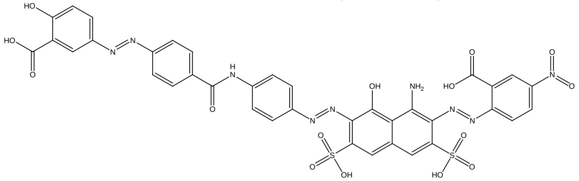	−3802.20	0.66	0.71	0.57

**115** [b]	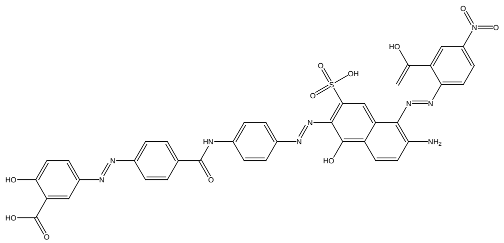	−3147.86	0.67	0.72	0.58
**116** [b]	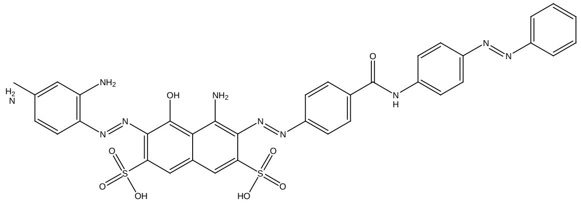	−3261.81	0.66	0.70	0.58
**117** [b]	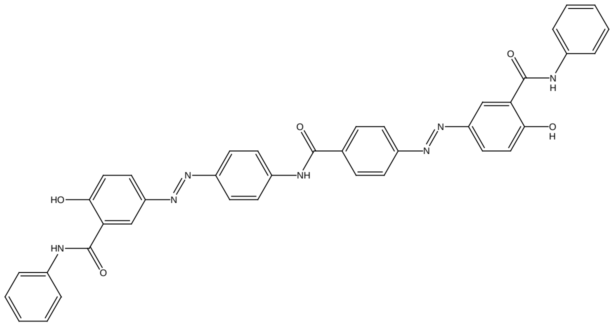	−2236.65	0.67	0.71	0.59
**118** [b]	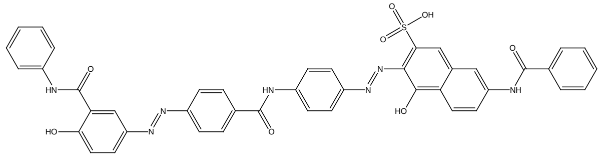	−3007.13	0.73	0.78	0.65
**119** [a]	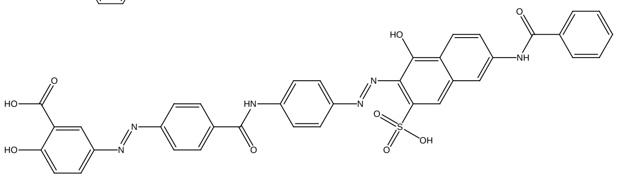	−2798.57	0.63	0.67	0.55

**120** [a]	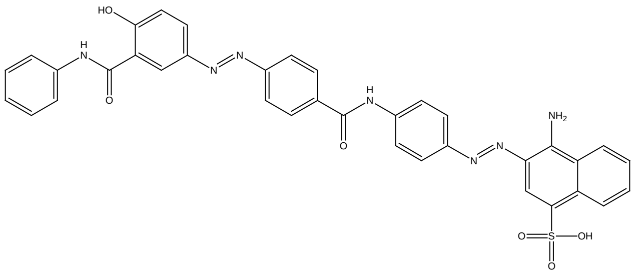	−2592.30	0.63	0.67	0.55
**121** [a]	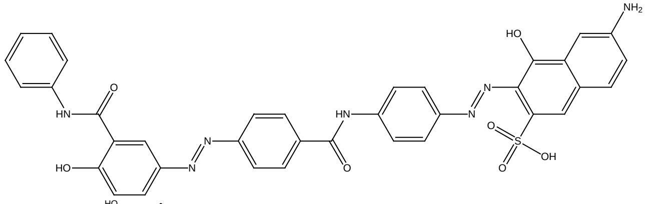	−2666.75	0.63	0.63	0.55
**122** [a]	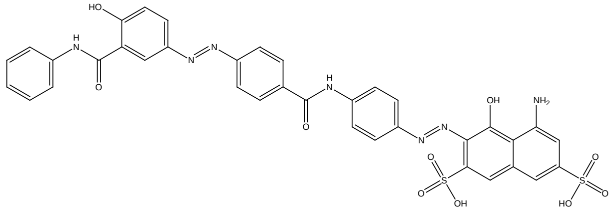	−3285.43	0.65	0.69	0.56
**123** [c]	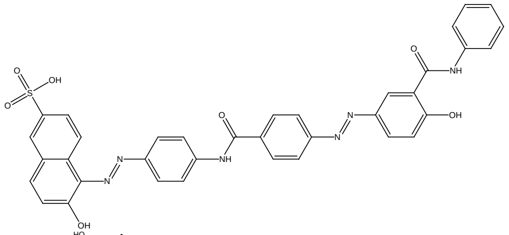	−2611.99	0.61	0.65	0.53
**124** [c]	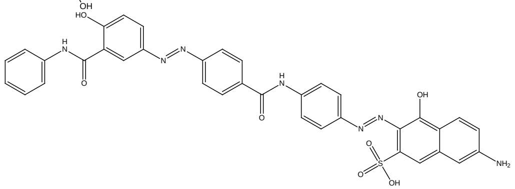	−2666.75	0.63	0.67	0.55

**125** [c]			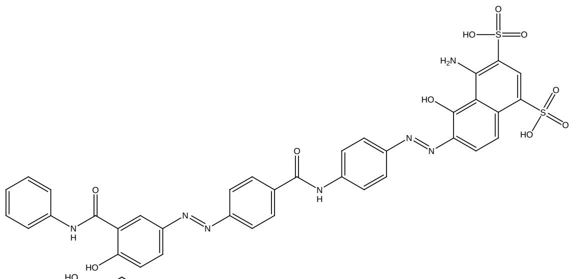	−3285.44	0.65	0.69	0.56
**126** [c]			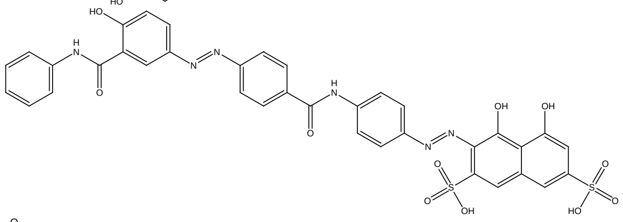	−3305.13	0.63	0.68	0.55
**127** [a]			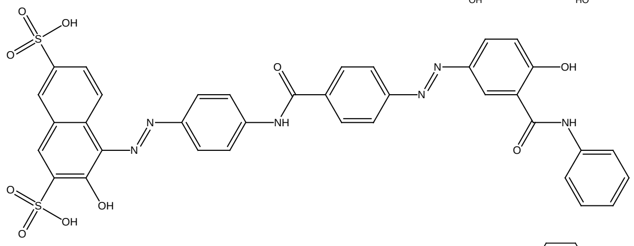	−3230.68	0.63	0.67	0.54
**128 *** [a]			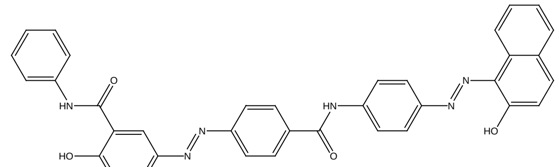	−1993.30	0.60	0.64	0.53
**129** [b]	25188-34-5		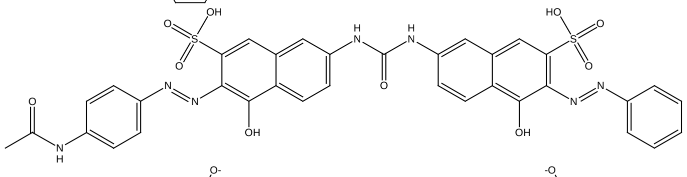	−3247.80	0.64	0.69	0.56
**130** [c]	3441-14-3	29160	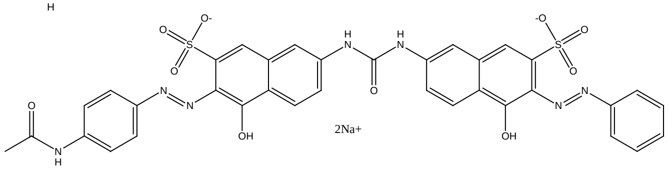	−3247.80	0.64	0.69	0.56

**131** [c]	25188-23-2	29150	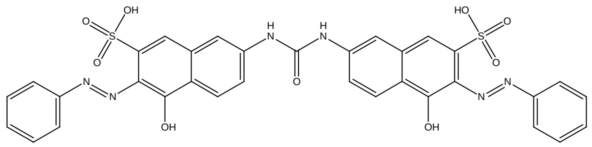	−3042.13	0.58	0.62	0.50
**132** [b]	2610108	35780	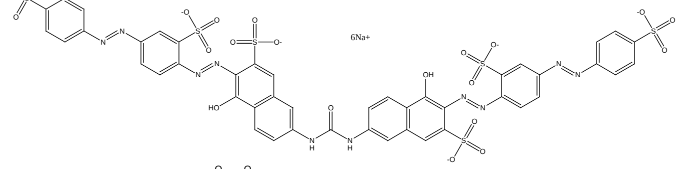	−6189.85	0.83	0.90	0.71
**133** [c]	2829-43-8		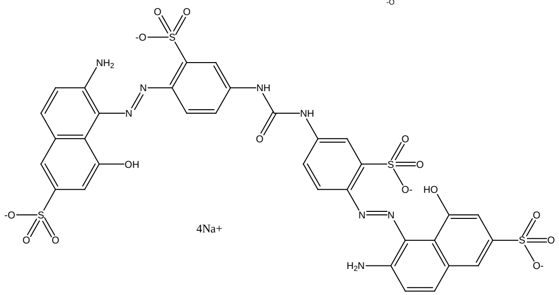	−4388.93	0.64	0.70	0.55
**134** [c]	3214-47-9	29025	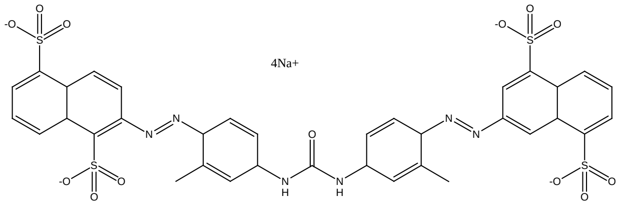	−4208.22	0.66	0.71	0.56
**135** [a]	14726-29-5	37155	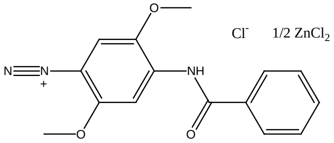	−958.48	0.30	0.31	0.25
**136** [a]	5486-84-0	37175	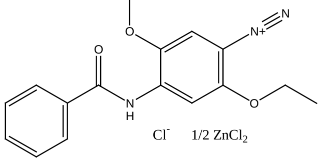	−1036.24	0.35	0.37	0.30

**137** [a]	49735-71-9	37125	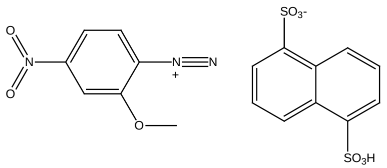	−652.59	0.30	0.15	0.10
**138**[c]	64071-89-9	37190	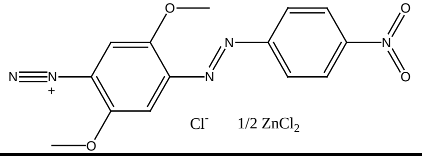	−1102.35	0.27	0.29	0.22

C.I. –Colour Index number, E(RHF/3-21G) – predicted SCF energy; ZPE – unscaled zero point correction; Hcorr – thermal correction to the enthalpy; Gcorr – thermal correction to the Gibbs free energy; * Examples of average experimental values, characterizing the reproducibility of the experimental measurements (in parentheses): for compound 2: 3.67 (3.52; 3.82); 4: 3.79 (3.88; 3.70); 5: 4.58 (4.56; 4.67; 4.50); 7: 3.32 (3.35; 3.29); 8: 3.31 (3.40; 3.21); 42: 3.62 (3.71; 3.57; 3.59); 43: 3.63 (3.63; 3.63); 57: 2.33 (2.39; 2.27); 58: 4.13 (4.20; 4.05); 74: 4.33 (4.41; 4.24); 82: 4.49 (4.60; 4.43; 4.44); 92: 3.79 (3.66; 3.92); 94: 4.30 (4.66; 3.93); 100: 4.55 (5.10; 4.20; 4.34); 103: 3.81 (3.86; 3.76); 128: 4.27 (4.14; 4.39); 134: 4.09 (4.20; 4.17; 3.89).

Table S2. Molecular properties of azo-, azo-anilide, diazonium salts derivatives.

No.	logP	AverPol	logS	HA	HD
**1**	4.22	23	−4.5	3	0
**2**	4.04	23	−4.17	4	1
**3**	3.31	29	−3.49	5	1
**4**	2.79	24	−3.77	5	1
**5**	3.52	27	−3.52	7	3
**6**	3.97	33	−3.97	5	1
**7**	3.73	24	−3.52	5	2
**8**	3.43	25	−3.37	6	3
**9**	4.04	27	−3.62	7	3
**10**	5.48	34	−4.85	5	2
**11**	5.48	34	−4.86	5	2
**12**	3.18	36	−4.68	6	1
**13**	3.17	36	−4.3	6	2
**14**	2.61	31	−3.85	6	1
**15**	3.33	35	−4.18	6	1
**16**	1.37	27	−3.36	6	2
**17**	0.89	28	−3.06	7	3
**18**	1.57	30	−3.35	8	3
**19**	−0.59	32	−3.65	9	3
**20**	4.53	26	−3.99	5	1
**21**	0.49	28	−3	8	4
**22**	3.77	22	−3.2	4	2
**23**	4.72	30	−4.43	5	2
**24**	4.72	30	−4.43	5	2
**25**	5.03	33	−4.33	7	3
**26**	4.20	31	−4.47	5	2
**27**	4.20	30	−4.46	5	2
**28**	2.19	32	−4.09	6	2
**29**	2.18	33	−4.04	6	2
**30**	2.45	36	−3.84	8	3
**31**	−0.72	38	−4.26	9	3
**32**	1.01	43	−4.53	11	3
**33**	5.07	27	−4.67	3	1
**34**	5.07	27	−4.66	3	1
**35**	6.09	31	−5.1	3	1
**36**	−0.01	38	−4.1	9	3
**37**	4.54	27	−4.83	3	1
**38**	1.21	42	−4.38	9	3
**39**	−0.64	38	−4.06	9	3
**40**	2.61	45	−3.88	10	5
**41**	−1.04	39	−3.66	10	4
**42**	−1.39	44	−4.06	10	4
**43**	−0.77	46	−4.19	10	4
**44**	0.06	42	−3.89	12	4
**45**	−0.27	43	−3.64	13	5
**46**	-2.18	45	−3.9	14	5
**47**	2.54	39	−4.13	11	4
**48**	2.08	35	−3.87	7	2

**49**	4.91	29	−4.72	4	1
**50**	4.95	31	−5.05	7	1
**51**	−2.17	50	−3.97	11	5
**52**	3.14	34	−3.67	7	3
**53**	5.58	29	−4.91	3	1
**54**	5.26	32	−4.94	4	1
**55**	−1.76	42	−3.3	12	4
**56**	3.32	38	−4.68	6	2
**57**	−1.98	50	−4.28	12	4
**58**	2.70	42	−4.38	9	3
**59**	0.33	44	−4.65	9	3
**60**	3.47	37		8	4
**61**	3.47	37	−3.93	8	4
**62**	5.01	44	−4.35	8	4
**63**	8.50	43	−5.31	5	1
**64**	8.50	43	−5.34	5	1
**65**	7.47	39	−5.17	5	1
**66**	9.01	45	−5.42	5	1
**67**	9.52	47	−5.5	5	1
**68**	1.63	50	−4.75	11	3
**69**	−3.88	61	−4.54	17	5
**70**	4.65	45	−4.78	8	2
**71**	7.60	43	−5.36	5	1
**72**	1.87	50	−4.74	11	3
**73**	2.87	64	−4.78	12	4
**74**	0.78	54	−4.37	14	4
**75**	−2.47	59	−4.67	17	3
**76**	0.78	53		14	2
**77**	6.33	115	−4.63	26	12
**78**	7.48	104	−4.73	24	10
**79**	5.02	66	−5.48	11	3
**80**	8.13	52	−5.37	6	2
**81**	6.60	60	−4.81	12	5
**82**	3.57	68	−5.41	12	4
**83**	5.95	77	−4.81	16	6
**84**	4.87	78	−4.69	17	5
**85**	4.98	75	−4.76	15	5
**86**	4.05	68	−5.18	12	4
**87**	4.14	65	−5.15	12	4
**88**	1.32	71	−4.76	14	2
**89**	−2.69	86	−4.9	20	8
**90**	1.13	86	−4.86	20	8
**91**	−0.27	88	−4.72	22	8
**92**	−4.13	87	−4.74	22	8
**93**	5.05	75	−4.73	15	5
**94**	2.98	27		4	0
**95**	4.51	73	−6.91	6	0
**96**	3.13	58	−4.62	12	4
**97**	2.11	60	−4.05	14	6
**98**	2.73	65	−4.43	16	6
**99**	3.94	67	−5.51	12	2

**100**	4.90	70	−5.43	12	4
**101**	5.42	77	−4.99	16	6
**102**	−1.87	83	−5.2	18	6
**103**	−0.54	95	−3.96	22	10
**104**	7.58	50	−4.69	10	5
**105**	6.54	70	−4.45	16	7
**106**	7.88	53	−4.88	11	5
**107**	6.66	64	−4.64	13	6
**108**	6.65	64	−4.64	13	6
**109**	2.89	71	−5.22	13	5
**110**	−1.16	82	−4.88	19	7
**111**	2.72	79	−4.48	18	8
**112**	0.91	77	−4.55	15	5
**113**	4.32	84	−4.55	20	7
**114**	3.69	87	−4.57	22	8
**115**	8.61	83	−4.75	18	7
**116**	5.39	80	−4.65	17	7
**117**	9.45	73	−5.34	9	5
**118**	7.65	86	−5.49	12	6
**119**	6.87	76	−5.09	13	6
**120**	6.04	73	−5.29	11	5
**121**	7.46	74	−5.06	12	6
**122**	4.65	79	−4.68	15	7
**123**	6.53	72	−5.16	11	5
**124**	7.46	74	−5.06	12	6
**125**	2.86	79	−4.69	15	7
**126**	3.33	79	−4.55	15	7
**127**	4.44	78	−4.7	14	6
**128**	9.35	66	−5.48	8	4
**129**	3.04	76	−4.56	14	7
**130**	3.04	76	−4.7	14	5
**131**	3.89	70	−4.54	13	6
**132**	9.20	116	−5.12	29	4
**133**	4.37	83	−4.87	21	6
**134**	−0.11	82	−5.47	17	2
**135**	2.59	29		4	1
**136**	3.30	33		4	1
**137**	1.32	25	−4.63	6	1
**138**	3.84	30		7	0

logP - the logarithm of the octanol-water partition coefficient; logS - the logarithm of solubility; AverPol - average polarizability; the number of donor (HD), respectively acceptor (HA) hydrogen bonds.

Table S3. Minimum energy structure of some azo- and azo-anilide derivatives (hydrogen bonds are expressed by dashed line).

No.	Structure
**25**	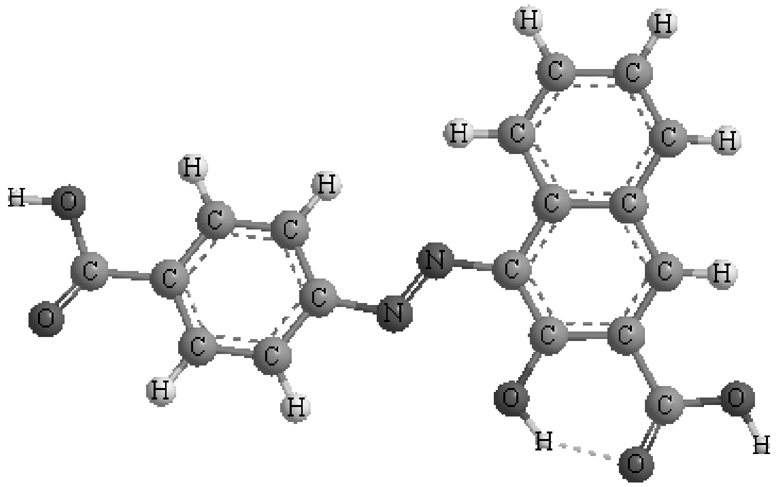
**26**	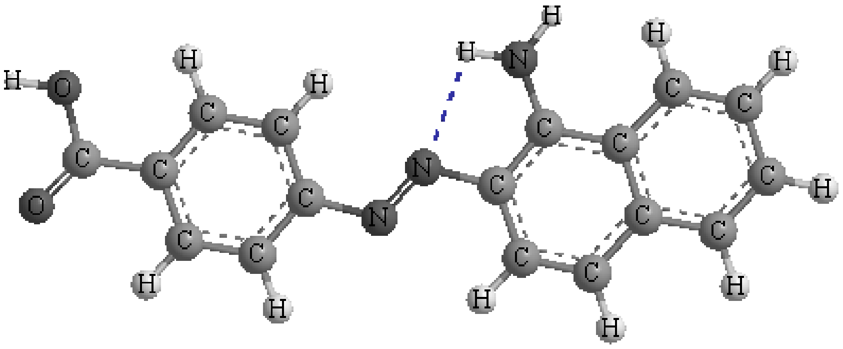
**39**	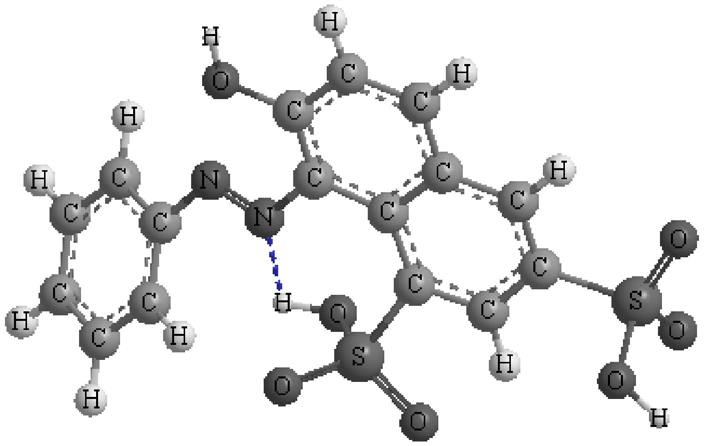
**58**	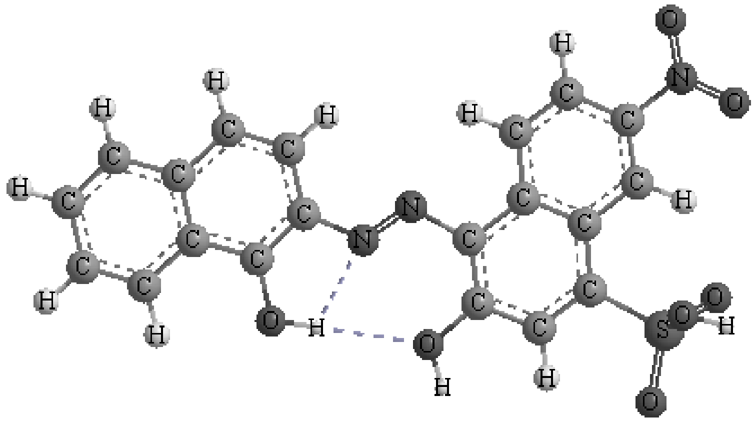

60

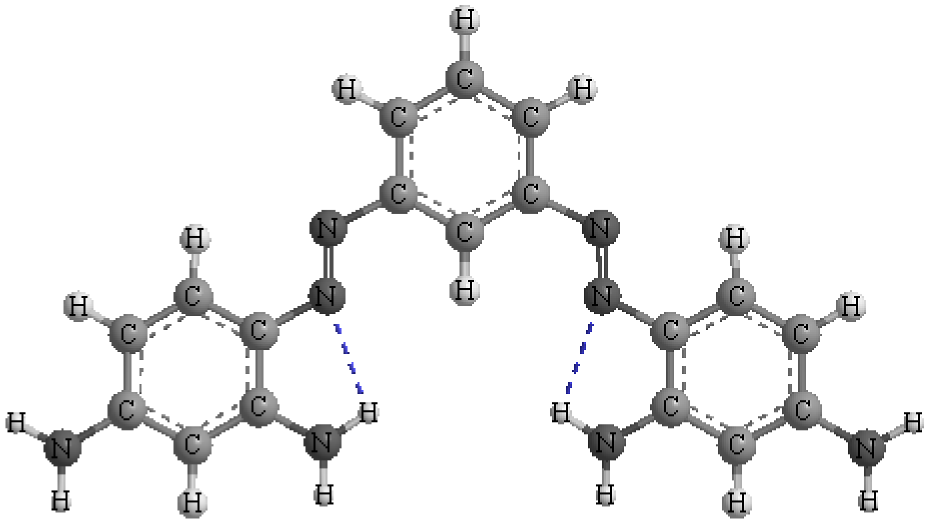

68

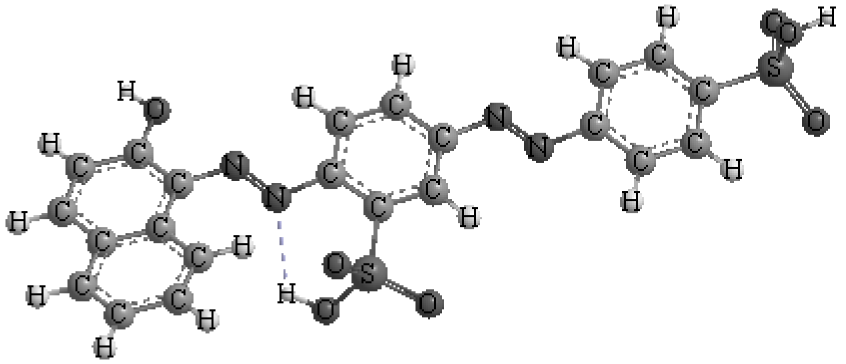

69

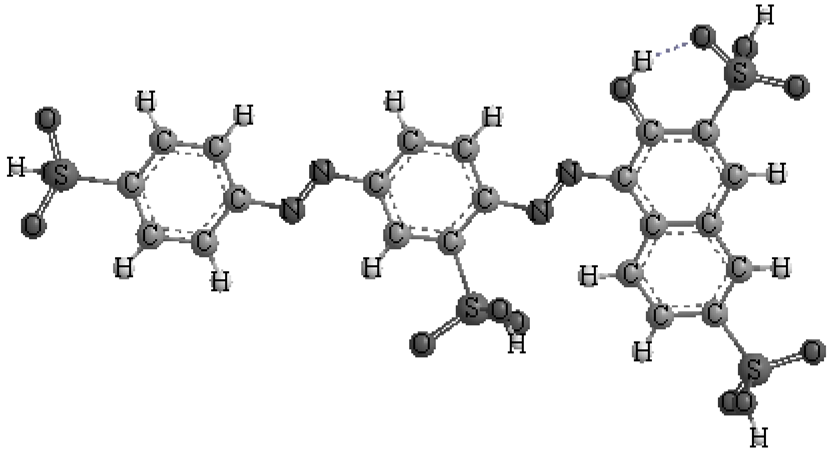

74

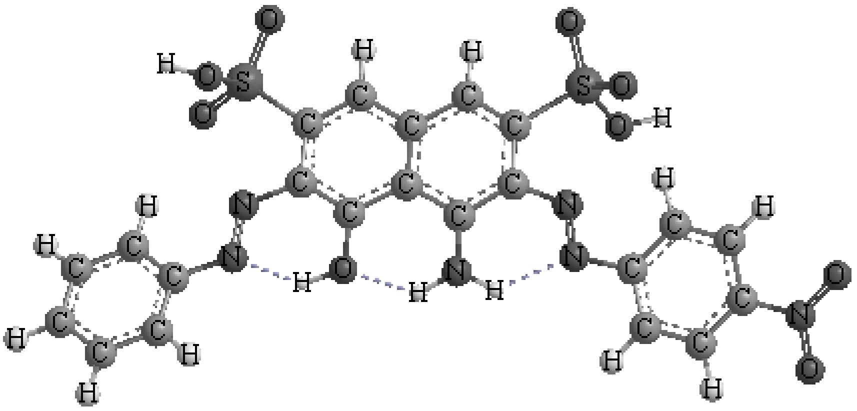

75

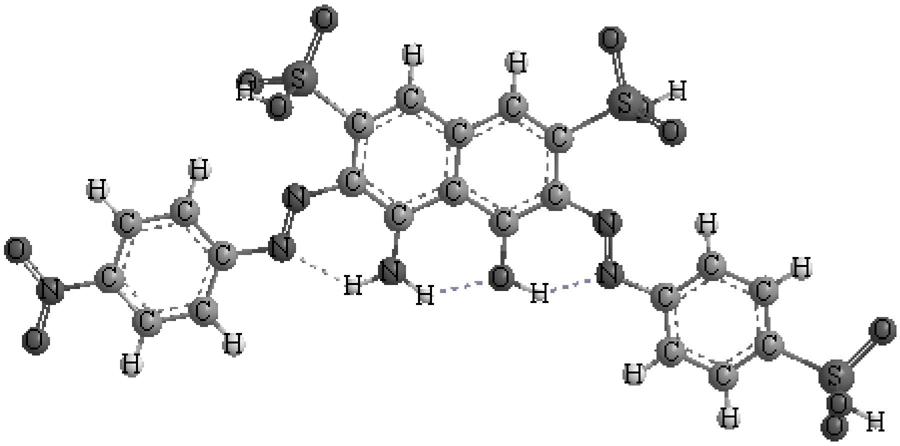

77

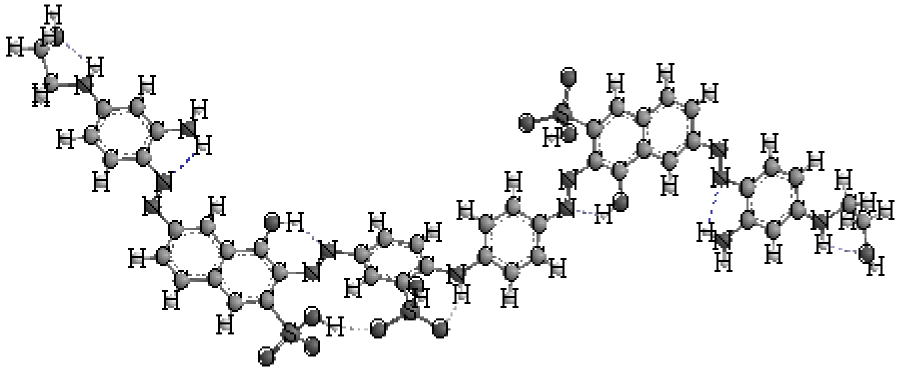

81

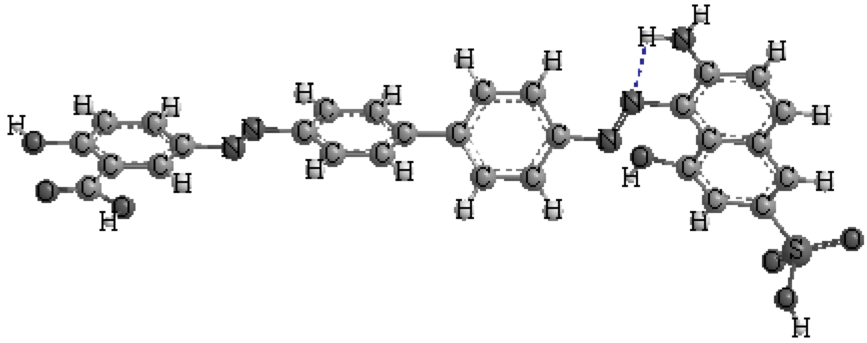

82

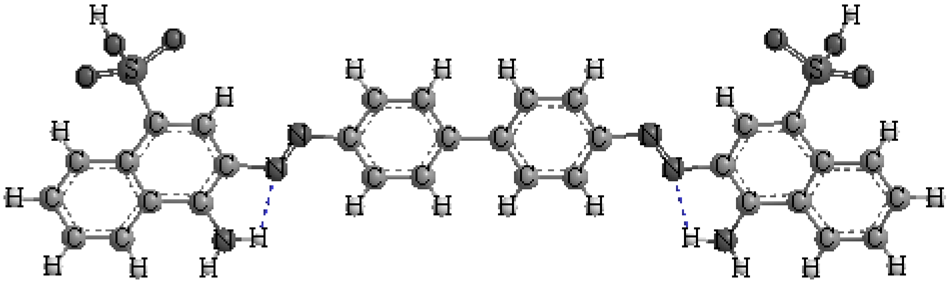

83

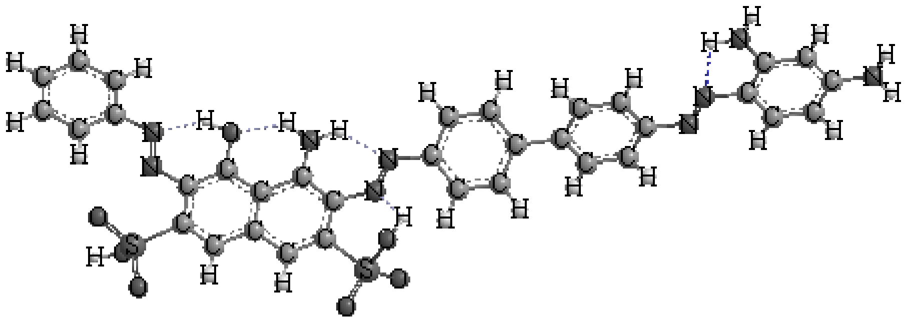

89

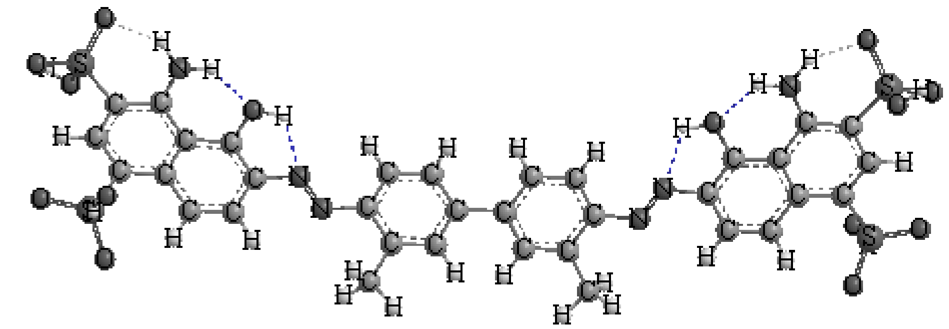

90

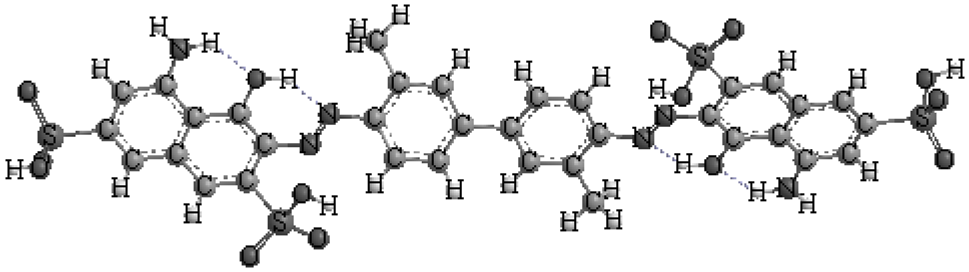

91

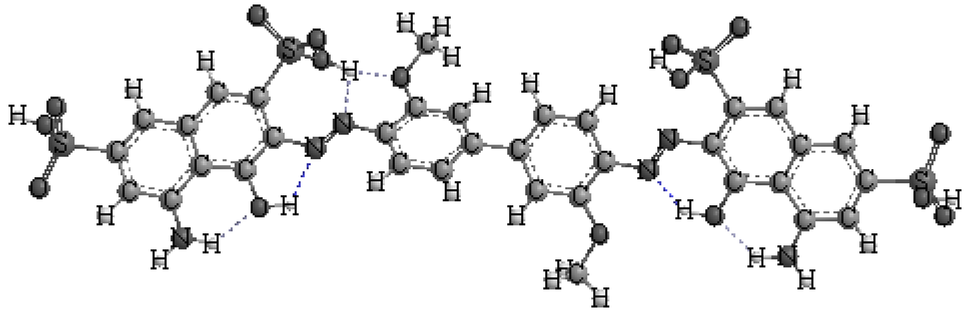

92

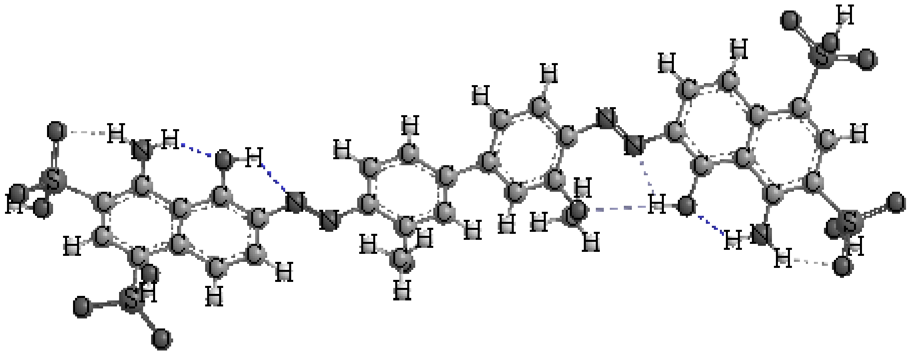

97

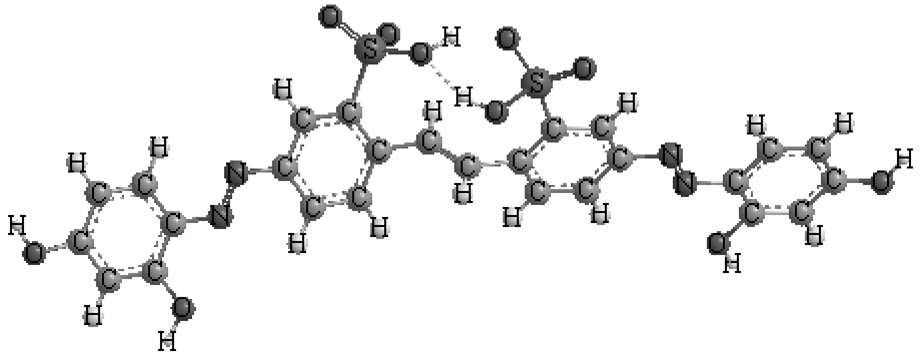

100

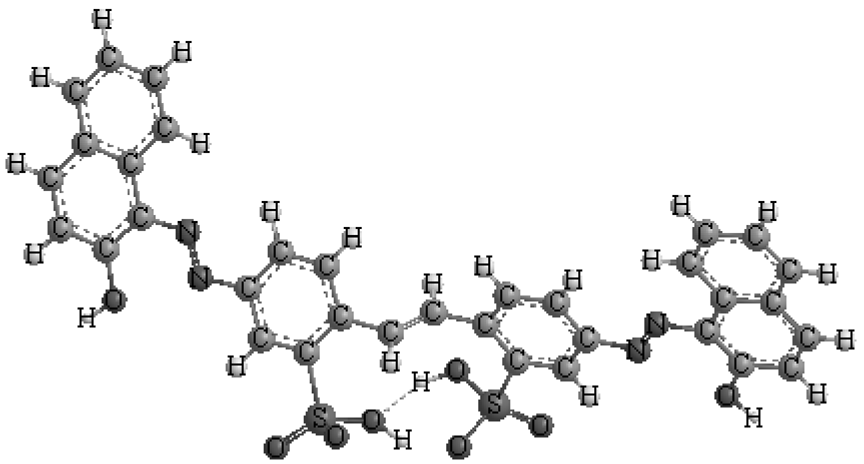

101

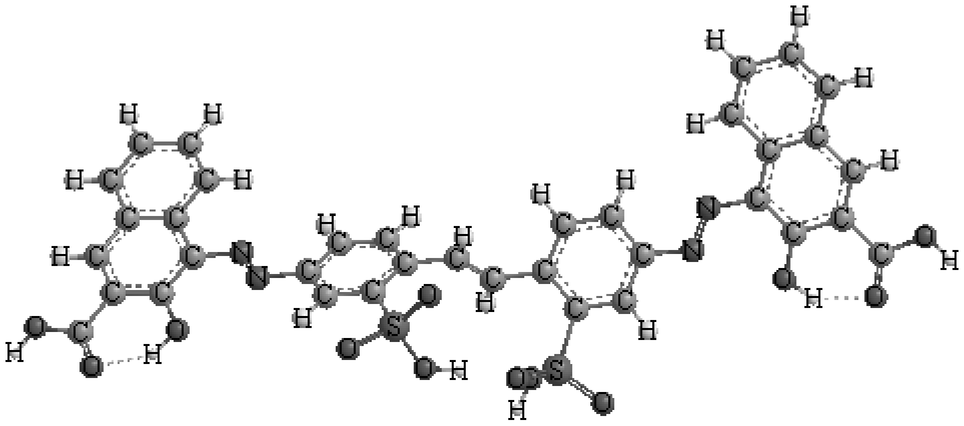

102

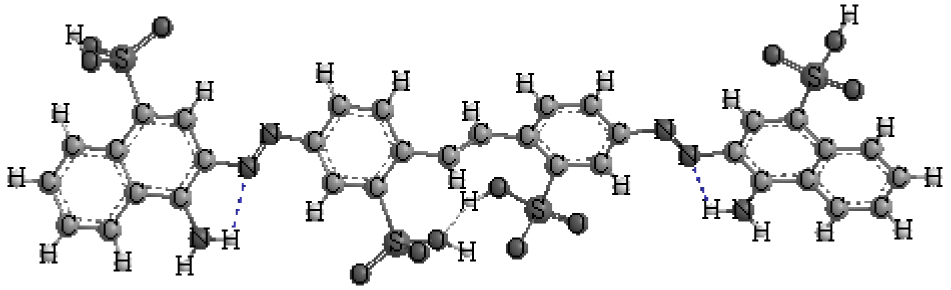

107

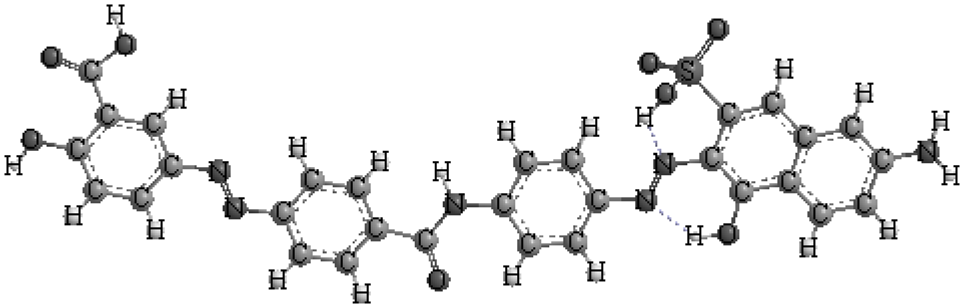

109

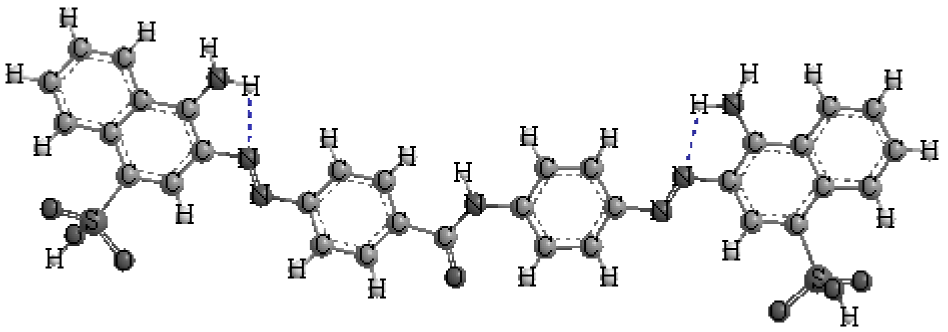

110

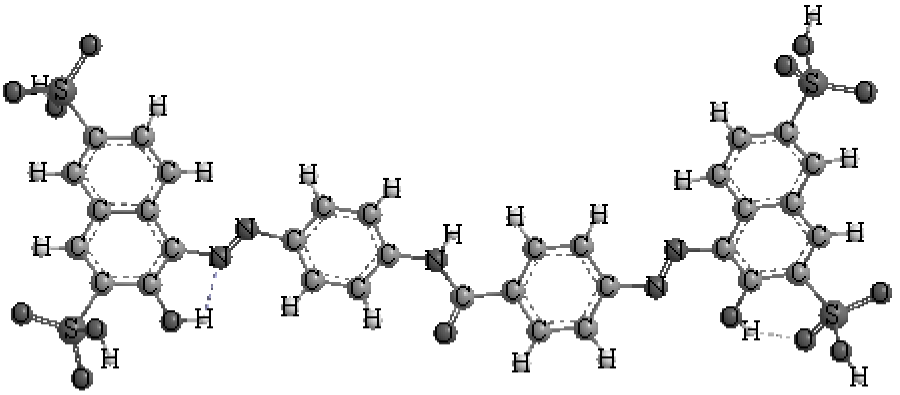

111

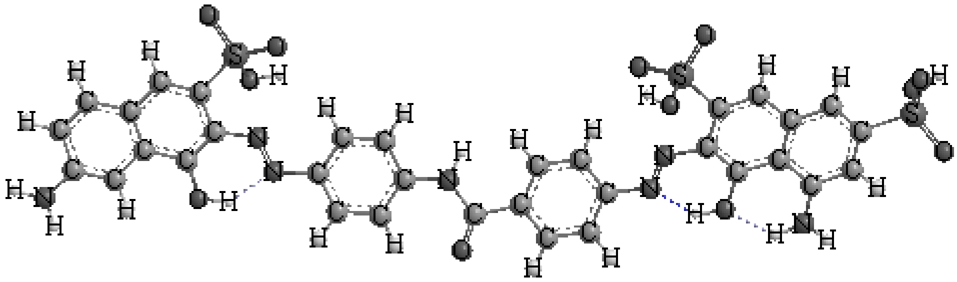

113

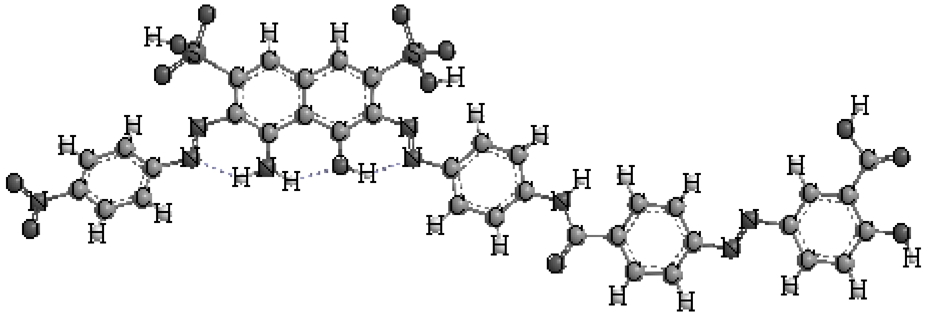

114

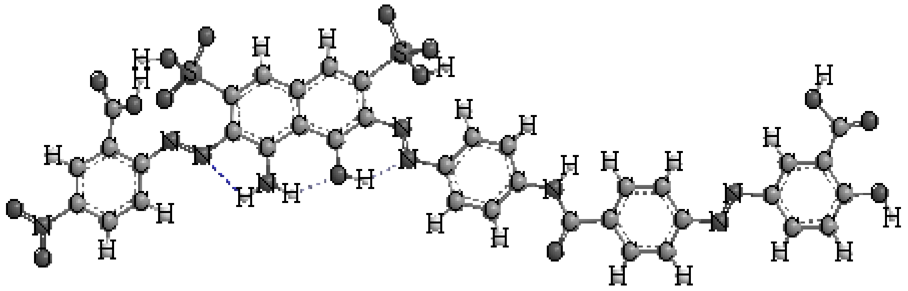

115

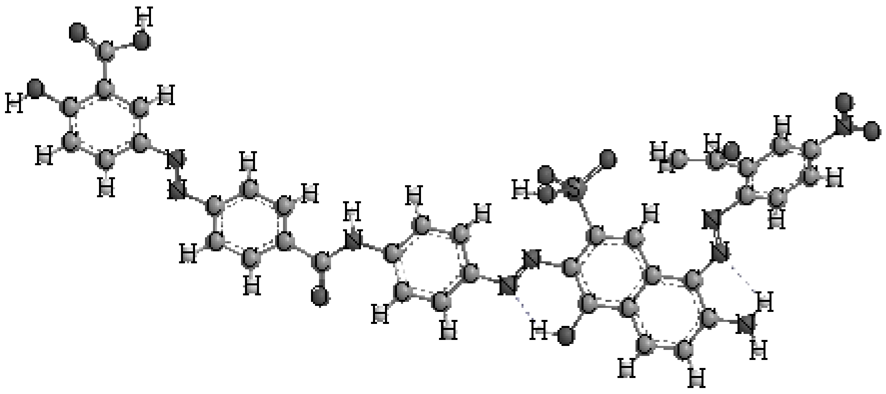

116

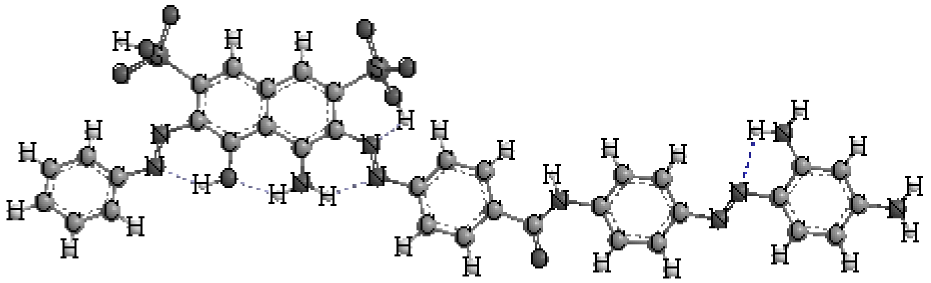

120

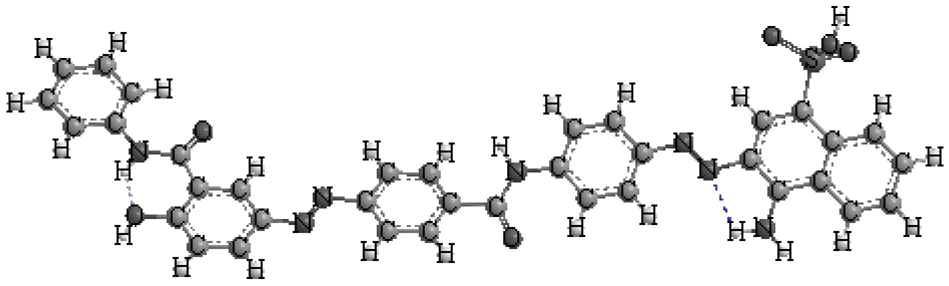

127

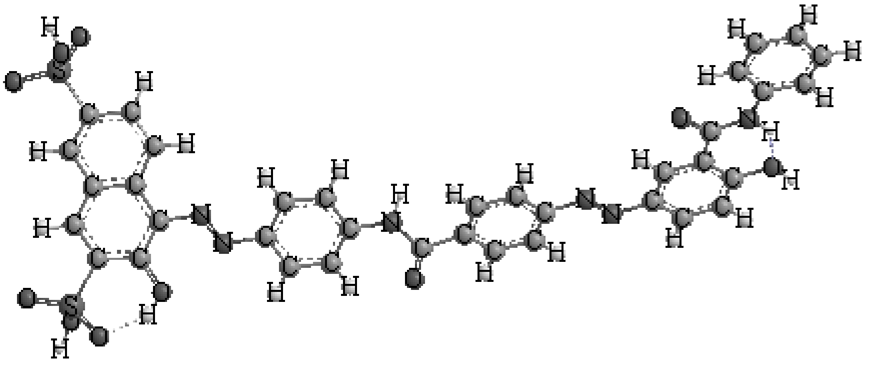

128

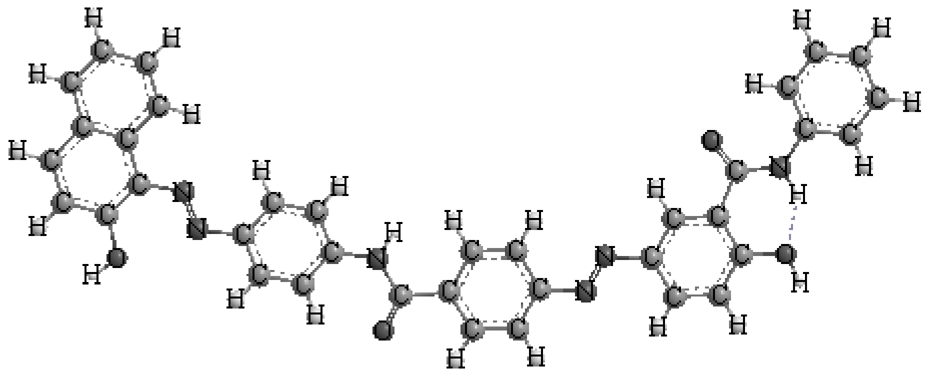

132

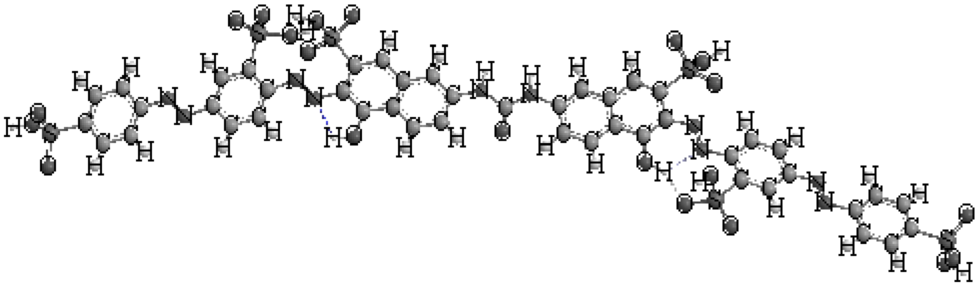

Supplement: Supplementary file 1 [file molecules-19-09798-s001.pdf]
